# Synthetic control of the surface area in nickel cobalt oxide for glucose detection via additive-assisted wet chemical method

**DOI:** 10.1038/s41598-022-20859-4

**Published:** 2022-11-15

**Authors:** Kyu-bong Jang, Kyoung Ryeol Park, Chan Bin Mo, Seongtak Kim, Jaeeun Jeon, Sung-chul Lim, Chisung Ahn, HyukSu Han, Dongju Kim, Seung Hwan Lee, Kang Min Kim, Sungwook Mhin

**Affiliations:** 1 Korea Institute of Industrial Technology, 137-41 Gwahakdanji-ro, Gangneung, 25440 Republic of Korea; 2 Korea Institute of Industrial Technology, 55, Jongga-ro, Jung-gu, Ulsan, 44413 Republic of Korea; 3 Korea Institute of Industrial Technology, 156 Gaetbeol-ro, Incheon, 21999 Republic of Korea; 4 Department of Energy Engineering, Konkuk University, 120 Neungdong-ro, Seoul, 05029 Republic of Korea; 5 Department of Advanced Materials Engineering, Kyonggi University, 154-42 Gwanggyosan-ro, Suwon, 16227 Republic of Korea; 6 School of Mechanical Engineering, Hanyang University, Seoul, 04763 Republic of Korea

**Keywords:** Catalyst synthesis, Electrocatalysis

## Abstract

We investigated the effect of specific surface area on the electrochemical properties of NiCo_2_O_4_ (NCO) for glucose detection. NCO nanomaterials with controlled specific surface areas were prepared by additive-assisted hydrothermal synthesis, and self-assembled nanostructures with urchin-, pine-needle-, tremella-, and flower-like morphologies were obtained. The novelty of this method is the systematic control of chemical reaction routes assisted by the addition of different additives during synthesis, which results in the spontaneous formation of various morphologies without any difference in the crystal structure and chemical states of the constituent elements. Such morphological control of NCO nanomaterials leads to considerable changes in the electrochemical performance for glucose detection. Combined with materials characterization, the relationship between the specific surface area and the electrochemical performance is discussed for glucose detection. This work can provide scientific insights for tailoring the surface area of nanostructures, which determines their functionality for potential applications in glucose biosensors.

## Introduction

Blood glucose levels provide vital information about human metabolism and physiological status^1,2^. For example, abnormal glucose levels in the body can be an important indicator of serious health problems, including diabetes, cardiovascular diseases, and obesity^3–5^. Thus, it is important to monitor blood glucose levels regularly to maintain good health. Although different types of glucose sensors using physicochemical detection have been reported, low sensitivity and slow response time are still impediments to continuous glucose monitoring systems^6–8^. In addition, the currently prevailing electrochemical glucose sensors based on enzymatic reactions have several limitations despite their advantages such as fast response, high sensitivity, and comparatively simple manufacturing procedure^9,10^. Accordingly, different types of non-enzymatic electrochemical sensors have been extensively studied to prevent enzyme denaturation, while maintaining the advantages of electrochemical biosensors^9,11–13^.

Transition metal-based compounds (TMCs) have sufficiently high catalytic activity toward glucose, expanding their applicability in electrochemical glucose sensors^13–15^. To date, various rational designs with facile synthetic methods for TMC have been suggested to further improve glucose detection in terms of sensitivity, selectivity, and electrochemical stability^16–18^. For example, single digit transition metal-based oxides such as copper oxide (CuO)^11,19^, zinc oxide (ZnO)^20^, nickel oxide (NiO)^21,22^, cobalt oxide (Co_3_O_4_)^23,24^, and cerium oxide (CeO_2_)^25^ present electrochemical activity towards glucose. Recent advance on the binary metal oxides such as nickel-cobaltite (NiCo_2_O_4_) for glucose detection show further synergetic effects on enhancing the electroactivity^26–30^. In particular, precise control of composition and morphology for the formation of TMCs with different nanostructures can effectively increase the detection sensitivity owing to their large surface areas, and thereby it is highly recommended to develop the TMCs with controlled morphologies for enhancing glucose detection^20,25,30–35^.

Herein, we report the NiCo_2_O_4_ (NCO) nanomaterials with various morphologies for glucose detection. The NCO nanomaterials were prepared by a simple hydrothermal method using various additives; chemical additives are one of the critical factors for self-assembly of nanostructures with various morphologies. We systematically investigated the effects of different morphologies of NCO on their electrochemical performance for glucose detection, including sensitivity, selectivity, low detection limits, and long-term stability.

## Result and discussion

**Figure 1:**
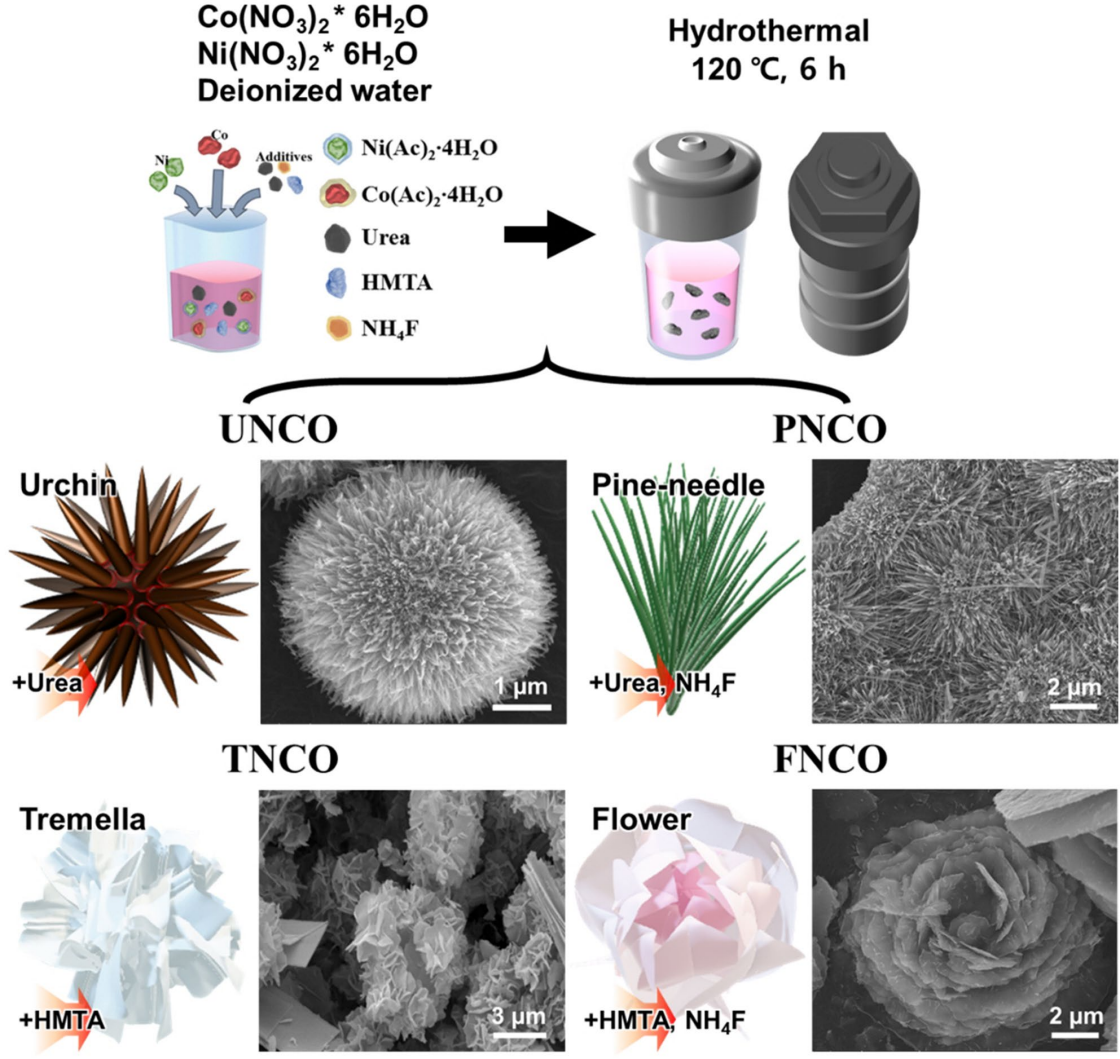
Schematic illustration of the formation process of morphology-controlled NiCo_2_O_4_ nanostructures. The schematic and SEM images of the different nanostructures are also shown.

**Figure 2:**
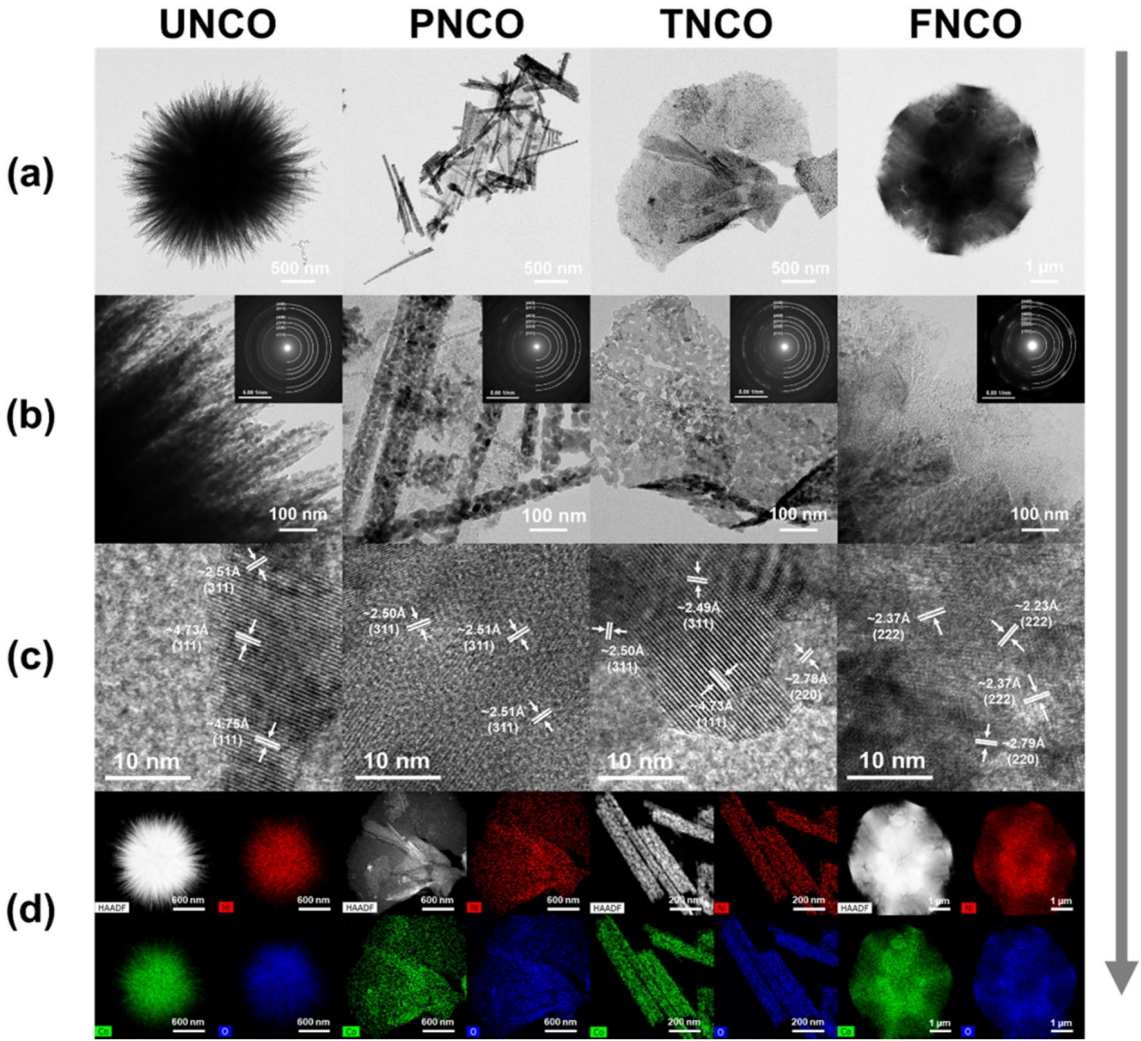
Morphological and structural characterizations of the NCO nanomaterials: (**a**) TEM images, (**b**) TEM images along with the SAED patterns, (**c**) lattice-resolved HRTEM images, and (**d**) corresponding HADDF images of Ni, Co, and O in the NCO nanomaterials.

We synthesized NCO nanomaterials with urchin-, pine-needle-, tremella-, and flower-like microstructures (abbreviated as UNCO, PNCO, TNCO, and FNCO, respectively). Figure 1 shows the distinct morphologies of UNCO, PNCO, TNCO, and FNCO. The SEM image and EDS mapping indicate that Ni, Co, and O are homogeneously distributed in the NCO nanomaterials, as shown in Figs. S1 and S2, respectively. Figure 2a,b show the representative TEM images of the NCO nanomaterials with distinguishable morphologies. UNCO is a self-assembled microsphere (diameter: ~ 5 µm) consisting of nanowires with NCO nanoparticles (average particle size: 20 nm). This unique microstructure is expected to provide a large surface area, promoting electrolyte diffusion as well as electron transfer. The addition of NH_4_F and urea during synthesis results in a thicker pine-needle-like microstructure (PNCO) with length of 3 µm and width of 60 nm consisting of larger nanoparticles. The addition of HMT instead of NH_4_F results in a tremella-like morphology (TNCO) with wrinkled nanosheets. The introduction of both NH_4_F and HMT during synthesis leads to aggregation of neighboring wrinkled nanosheets, resulting in the formation of a flower-like morphology (FNCO). The HRTEM images (Fig. 2c) reveal clear lattice fringes with inter-planar spacings of 0.473, 0.278, 0.50, and 0.237 nm, which corresponds to the (111), (220), (311), and (222) planes of NiCo_2_O_4_, respectively^27^. The selected area electron diffraction (SAED) patterns of the NCO nanomaterials (insets of Fig. 2b) also confirm the polycrystalline nature of NiCo_2_O_4_. The results of high-angle annular dark-field (HAADF) imaging and EDS mapping indicate that all the elements are homogeneously distributed in the NCO nanomaterials, as shown in Fig. 2d.

**Figure 3:**
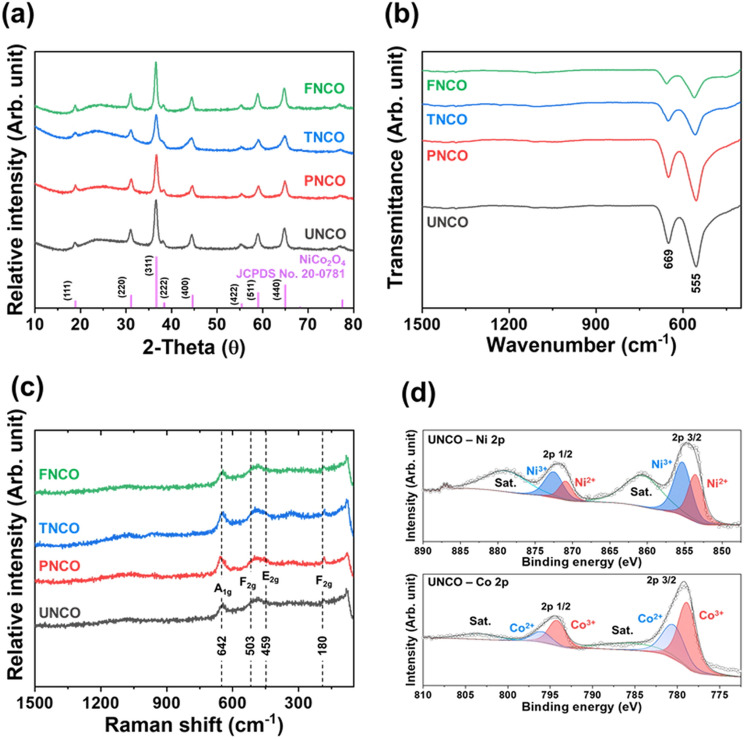
(**a**) XRD patterns, (**b**) FT-IR spectra, and (**c**) Raman spectra of the NCO nanomaterials; (**d**) XPS spectra of Ni 2p and Co 2p of UNCO.

The XRD patterns of the NCO nanomaterials with various morphologies are shown in Fig. 3a. The diffraction peaks at 18.9, 31.1, 36.6, 44.6, 59.1, and 64.9° are indexed to the (111), (220), (311), (400), (511), and (440) planes, respectively of NiCo_2_O_4_ with a cubic spinel structure (JCPDS No. 20-0781)^36^. The FT-IR spectra of the NCO nanomaterials are shown in Fig. 3b. The two strong vibrational bands in the region between 555 and 669 cm^−1^ correspond to metal (Ni and Co)–oxygen stretching from tetrahedral and octahedral sites, respectively of spinel NiCo_2_O_4_^37^. To further understand the structural properties of the NCO nanomaterials, Raman spectra were acquired, as shown in Fig. 3c. The four peaks observed at 180, 459, 503, and 642 cm^−1^ correspond to the Raman-active F2g, E2g, F2g, and A1g modes, respectively of spinel NiCo_2_O_4_. XPS measurements were performed to identify the surface chemical states of the elements in the NCO nanomaterials. Figure 3d shows the XPS spectra of UNCO. The Ni 2p spectra shows two major peaks located at binding energies of 854.8 and 872.3 eV corresponding to Ni 2p_3/2_ and Ni 2p_1/2_ with two shakeup satellites at 860.6 and 879.1 eV, respectively. This indicates the presence of Ni^2+^ and Ni^3+^ oxidation states in NCO. The peaks at approximately 855.9 and 873.4 eV are ascribed to Ni^3+^, and the peaks located at around 854.2 and 871.6 eV are assigned to Ni^2+^. Similarly, the Co2p spectra of the two spin orbit doublets reveal the characteristic peaks of Co^2+^ and Co^3+^ at 780.4 (Co 2p_3/2_) and 795.7 eV (Co 2p_1/2_). The peaks at 796.0 and 780.3 eV are assigned to Co^2+^, while those at 794.4 and 779.3 eV correspond to Co^3+^. It is to be noted that the multi-valence states (Ni^2+^/Ni^3+^ and Co^2+^/Co^3+^) of metal ions in NiCo_2_O_4_ are beneficial for improving the electrochemical activity^37,38^. The Ni2p and Co2p spectra of UNCO, PNCO, TNCO, and FNCO show similar results, as shown in Fig. S3. In addition, the O1s spectra of all the NCO nanomaterials (Fig. S4) show two peaks at 592.4 and 531.2 eV, associated with typical metal–oxygen bonds and oxygen in hydroxyl groups on the surface of NCO, respectively^39^. Despite the structural similarities of the NCO nanomaterials, the morphological differences in the additives suggest that each additive can participate in the chemical reaction differently for the formation of NCO. This controls the energetically preferred steps for nucleation and grain growth, thus tuning the particle size and degree of agglomeration. In this manner, the control of different process parameters, including additives, reaction time, and temperature during synthesis, can be used to engineer the microstructure and improve the electrochemical performance of the NCO nanomaterials for glucose detection.

Tailoring the morphologies of NCO nanomaterials is closely related to the formation of initial phases originated from different additives depicted in Fig. S5. Furthermore, both the XRD patterns and the Raman spectra of the as-prepared samples (Figs. S6 and S7a) reveal that the participation of different chemical additives results in crystallographic differences: Ni and Co carbonate hydroxide were mainly observed in the urchin- and pine-needle-like structures, while the tremella- and flower-like structures show the presence of Ni and Co hydroxide. The FT-IR and XPS spectra of the as-prepared samples as shown in Figs. S7b to S9 also provide clear evidence for the aforementioned crystallographic differences. From the materials characterization of the as-prepared samples, it is evident that the additives participate in the hydrothermal reaction and provide different reaction pathways to prepare initial phases with various morphologies^40–42^. The self-assembly of various morphologies, consisting of one-dimensional (1D) nanowires and two-dimensional (2D) nanosheets, is attributed to the different chemical states of the initial phases (chemical states of Ni and Co ions, and functional groups) with subsequent crystal growth^42–47^. During the post-heat treatment, different initial phases are transformed to spinel NCO while maintaining their unique morphologies, as confirmed by Figs. 2 and 3a.

**Figure 4:**
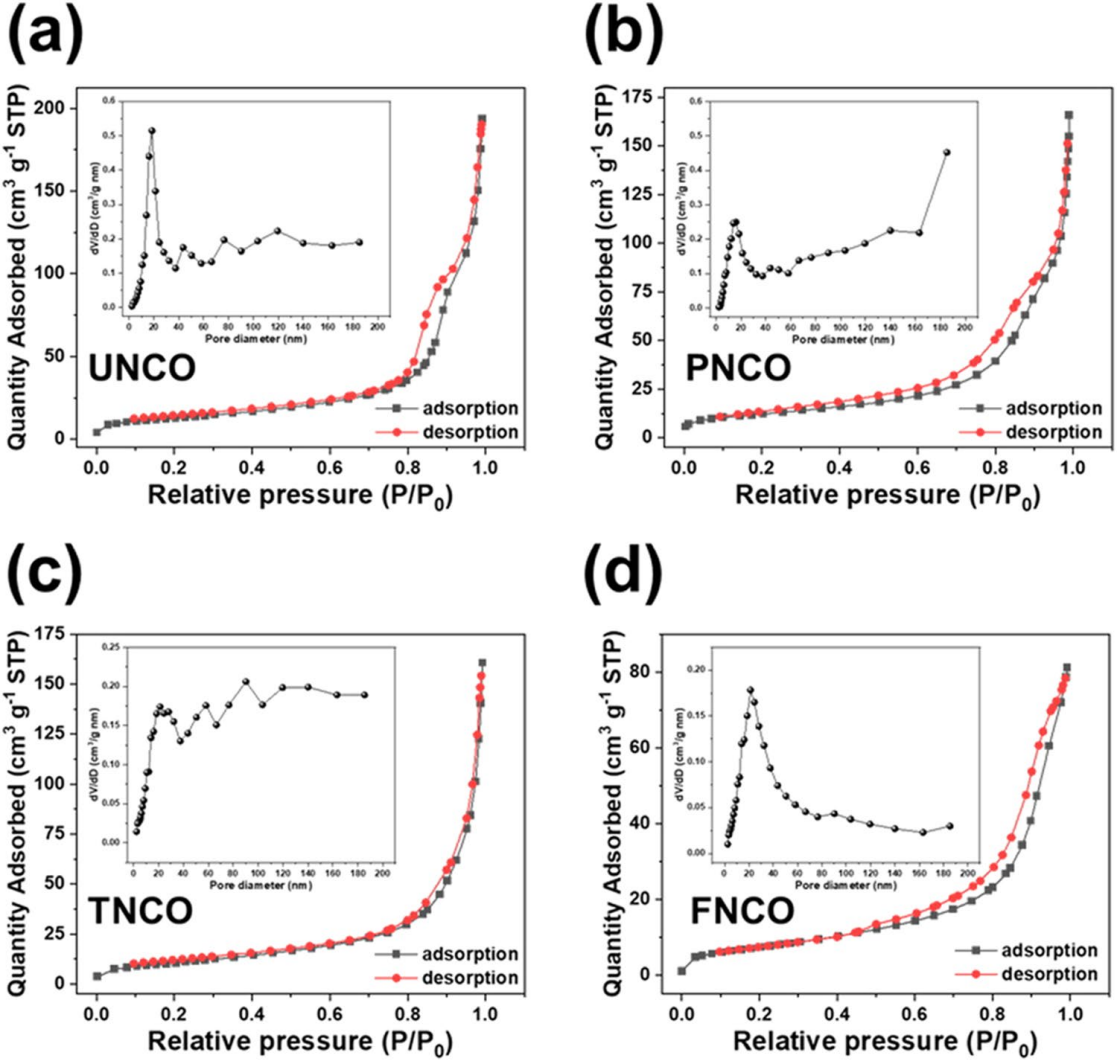
BET results of (**a**) UNCO, (**b**) PNCO, (**c**) TNCO, and (**d**) FNCO. The insets show the corresponding pore size distributions.

The morphological differences in the NCO nanomaterials can affect the electrochemically active surface area for glucose detection, which determines the overall electrochemical performance of the glucose sensor. The N_2_ adsorption–desorption isotherm of BET was used to evaluate the pore size and specific area of the NCO nanomaterials. Figure 4 shows the BET isotherms of the different NCO nanomaterials. The BET specific area values of UNCO, PNCO, TNCO, and FNCO were evaluated to be 45.303, 43.304, 38.861, and 27.260 m^2^ g^−1^, respectively. UNCO exhibits the highest BET specific area (45.303 m^2^ g^−1^) and the largest pore volume (0.2849 cm^3^ g^−1^) coupled with narrow pore size distribution. The BET results of the NCO nanomaterials are summarized in Table 1. The N_2_ adsorption–desorption curves closely resemble the type-IV isotherm hysteresis loop, which implies that all the samples have a mesoporous structure^48^. It is expected that mesoporous UNCO with the highest surface area and largest pore volume can provide abundant active sites for redox reactions, thus promoting electrochemical performance.

**Table 1:** Summary of the BET results of the NCO nanomaterials.

Material	Sample	BET surface area (m^2^/g)	Average pore size (nm)	BJH cumulative volume of pores (cm^3^/g)
NiCo_2_O_4_	UNCO	45.303	25.099	0.2849
	PNCO	43.304	23.319	0.2528
	TNCO	38.861	23.761	0.2315
	FNCO	27.260	18.006	0.1244

**Figure 5:**
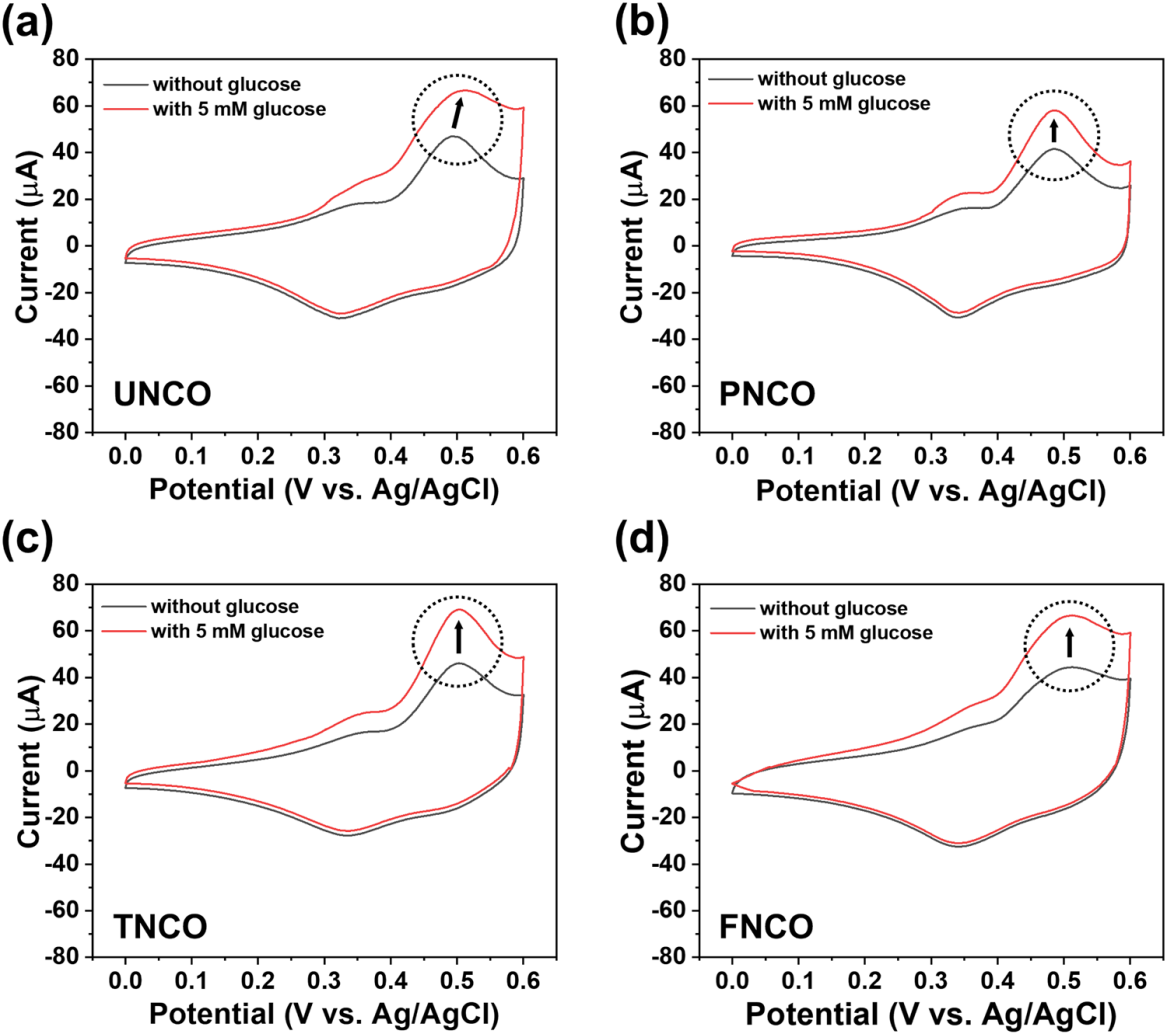
CV curves of (**a**) UNCO, (**b**) PNCO, (**c**) TNCO, and (**d**) FNCO electrodes in the absence of glucose and with the addition of 5 mM glucose at a scan rate of 50 mVs^−1^.

The electrochemical redox reactions of the NCO nanomaterials with different morphologies for glucose detection were evaluated by CV measurements. Figure 5 shows the CV curves of the NCO nanomaterials in 0.1 M NaOH alkaline electrolyte with and without 5 mM glucose at a scan rate of 50 mVs^−1^. The redox peaks were observed in the absence of glucose at 0.50 and 0.35 V, corresponding to the redox reactions related to M–O (M: Ni^2+^, Co^2+^) and M*-O-OH (M*: Ni^3+^, Co^3+^) with the aid of OH^−^ anion^49^. With the addition of 5 mM glucose, a significantly increased redox reaction occurs on the surface of NCO nanomaterials, which may be attributed to the oxidation of glucose to gluconolactone. Figure S10 shows the dependence of redox peak currents at scan rates of 5–100 mV s^−1^ in 0.1 M NaOH solution. It is evident that the redox peak currents increase with an increase in the scan rate, which suggests similar diffusion-controlled electrochemical behaviors of the NCO nanomaterials^50,51^. As shown in Fig. S11, electrochemical surface area (ECSA) of the UNCO, PNCO, TNCO, and FNCO were evaluated to be 2.15, 1.47, 1.2, and 1.03 cm^2^, respectively. This reveals that UNCO is beneficial to the electrocatalytic process to promote glucose detection.

**Figure 6:**
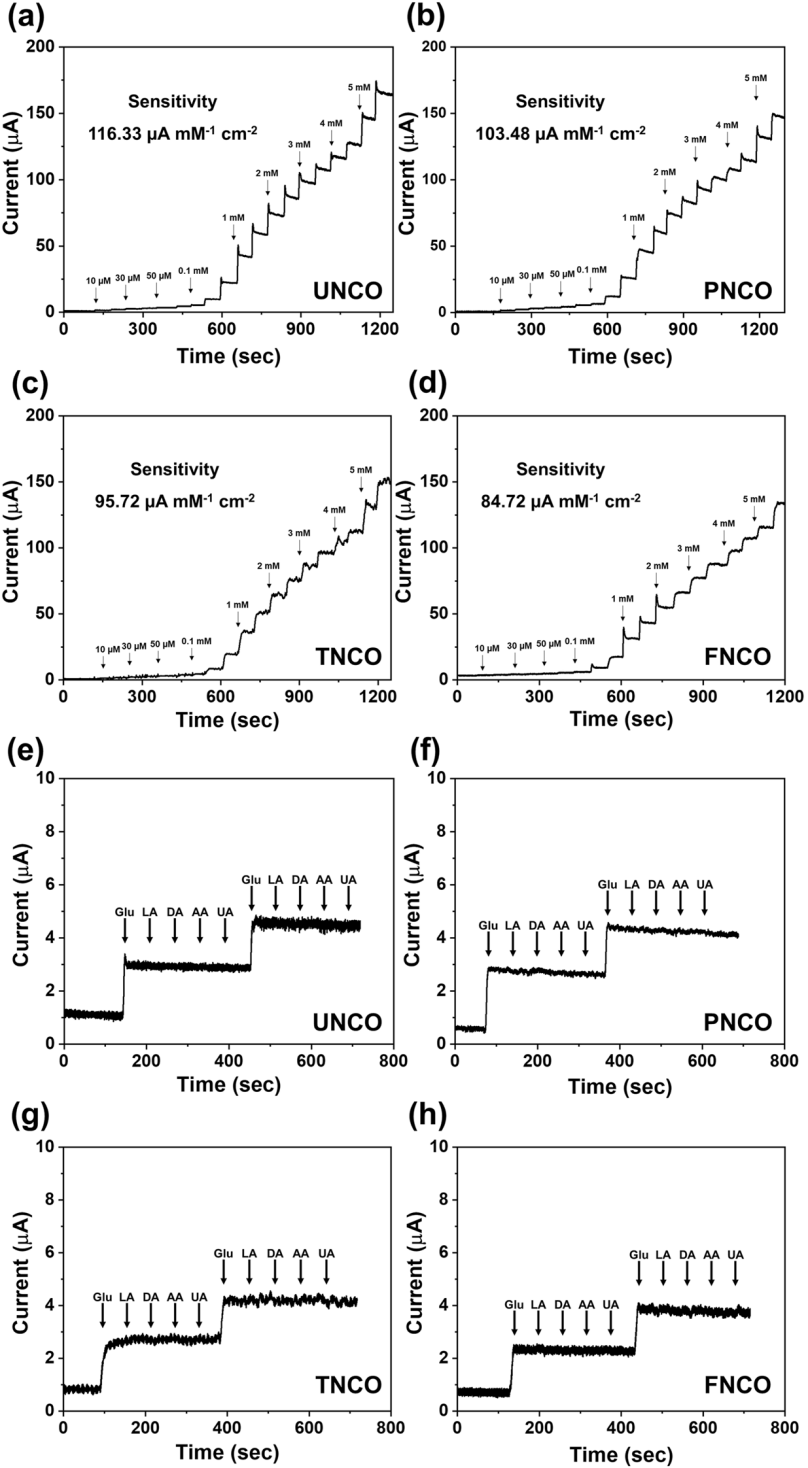
CA responses of (**a**) UNCO, (**b**) PNCO, (**c**) TNCO, and (**d**) FNCO electrodes with the addition of glucose to 0.1 M NaOH solution at 0.50 V. The inset shows the calibration curves for the current response of the NCO nanomaterials; CA responses of (**e**) UNCO, (**f**) PNCO, (**g**) TNCO, and (**h**) FNCO with the stepwise addition of 1 mM glucose and 0.1 mM interfering species (LA, DA, AA, and UA).

The electrochemical performance of the NCO nanomaterials for glucose detection was investigated, and the results are shown in Fig. 6. The glucose sensitivity was evaluated by the CA method, which was performed by step-wise addition of glucose at different concentrations (0.01–6 mM) in 0.1 M NaOH solution at intervals of 60 s under 0.5 V. As shown in Fig. 6a–d, the NCO nanomaterials show different sensitivities between 84.72 and 116.33 μA mM^−1^ cm^−2^ with a high correlation coefficient (R^2^) between 0.99 and 0.993. The calibration curve between glucose concentration and current response of the NCO nanomaterials is presented in Fig. S12. The calculated limits of detection (LOD) of the NCO nanomaterials are in the range 0.0623–0.0783 µM. Based on the results of the CA test, UNCO shows the highest sensitivity (116.33 μA mM^−1^ cm^−2^) over a wide detection range. This can be attributed to its unique urchin-like morphology consisting of a mesoporous structure with a large specific surface area, providing more abundant active sites for glucose species. The electrochemical performances of the NCO nanomaterials, as summarized in Table S1, establish the excellent electrochemical glucose detection properties of the NCO nanomaterials prepared in this study.

The anti-interference ability of glucose detection is another important factor for selective and sensitive detection of glucose with interfering compounds. Figure 6e–h show the anti-interference ability of the NCO nanomaterials in 0.1 M NaOH solution. General interfering molecules such as LA, DA, AA, and UA were selected and added to the electrolyte. The current response of the NCO nanomaterials to glucose is evident. However, there is no change in the current response to UA, DA, AA, and LA, which implies that NCO nanomaterials have excellent selectivity for glucose detection, regardless of their morphological differences. Figure S13 shows the stability of the NCO nanomaterials as examined by CA response in 0.1 M NaOH, where 1 mM glucose was added to the electrolyte for an extended period of time (80,000 s). The current responses of UNCO, PNCO, TNCO, and FNCO are 98.6, 97.5, 98.4, and 96.8%, respectively of the initial current upon addition of another 1 mM glucose after 80,000 s. All the NCO nanomaterials exhibited stable redox reactions for glucose species over extended durations. Especially, current signal of the UNCO retains not only 97.1% of its initial current, but also morphology and chemical bonding nature after long-term stability test for 7 days under ambient conditions (Figs. S14 and S15a). Also, the reproducibility and repeatability of the UNCO were examined as shown in Fig. S15b,c. The relative standard deviation (RSD) of the reproducibility and repeatability were calculated as 2.42 and 2.14%, respectively, which shows potential application as the glucose sensor in industrial level. It is suggested that the UNCO has excellent structural and chemical stability under oxidizing condition for glucose detection.

It is clear that the electrochemical performance of the NCO nanomaterials for glucose detection is mainly attributed to the structural merits of the initial phases prepared by the additive-assisted hydrothermal method (Fig. S16). UNCO with a large surface area possesses more abundant electroactive sites than the other nanostructures, which is helpful for improving the redox reaction between the active materials and glucose species. The mesoporous structure of UNCO can easily expose more Ni and Co sites to the electrolyte for glucose detection, leading to a rapid electrochemical response. The 1D nanowires in UNCO can further boost the diffusion rate by providing shorter transport pathways for ions and electrons. Owing to the aforementioned unique structural features, the electrochemical performance of UNCO for glucose detection is superior to that of PNCO, TNCO, and FNCO. It is suggested that the unique morphology of UNCO within the highest surface area and pore size can provide superior electrochemical performance for glucose detection.

## Conclusion

The effect of specific surface area on the electrochemical performance of NCO nanomaterials was investigated. NCO nanomaterials with different specific surface areas were prepared by a facile hydrothermal method with different additives. The different additives underwent different chemical reactions during synthesis, forming different initial phases. This conduced to the self-assembly of various nanostructures with urchin-, pine-needle-, tremella-, and flower-like morphologies. Subsequent post-heating resulted in similar chemical states of crystalline NCO nanomaterials with a spinel structure, while maintaining their unique morphologies. Depending on the surface area of the different morphologies, the electrochemical performance of the NCO nanomaterials for glucose detection was considerably improved. Especially, glucose sensitivity of NCO nanomaterials with urchin-like morphology increased up to 116.33 μA mM^−1^ cm^−2^ with high correlation coefficient (R^2^) of 0.99 in the linear range 0.01–6 mM. This work can provide a scientific basis for morphology engineering to tune the specific surface area, which can further enhance the electrochemical performance for non-enzymatic biosensor applications.

## Experimental details

### Materials and reagents

Ni(NO_3_)_2_·6H_2_O, Co(NO_3_)_2_·6H_2_O, urea, hexamethylene-tetramine (HMT), ammonium fluoride (NH_4_F), sodium hydroxide (NaOH), d-( +)-glucose, lactic acid (LA), dopamine hydrochloride (DA), l-Ascorbic acid (AA), and uric acid (UA) were purchased from Sigma-Aldrich. All of the reagents used were of analytical grade and used as received without further purification.

### Synthesis of the morphology-controlled NiCo_2_O_4_

NiCo_2_O_4_ was synthesized via a simple hydrothermal method followed by post-heat treatment. Briefly, 1 mmol of nickel nitrate (Ni(NO_3_)_2_∙6H_2_O) and 2 mmol of cobalt nitrate (Co(NO_3_)_2_∙6H_2_O) were dissolved in 30 mL of distilled water. To control the morphology of NiCo_2_O_4_, additives such as urea, ammonium fluoride, and hexamethylene-tetramine (HMT) were selectively added to the above solution. Then, the whole mixture was transferred to a 50 mL Teflon-lined autoclave vessel and subjected to hydrothermal reaction at 120 °C for 6 h in a convection oven. After naturally cooling to room temperature, the obtained precipitate was washed with distilled water and ethanol several times by centrifugation and then dried at 60 °C overnight. Subsequently, the as-prepared sample was calcined at 400 °C for 4 h in an ambient atmosphere. The details of the experiments are presented in Table S2 of Supporting Information.

### Material characterizations

X-ray diffraction analysis (XRD, X’Pert-Pro MPD; PANalytical) with Cu-Kα radiation (λ = 0.15418 nm) at 40 kV and 30 mA was performed to study the structural properties of all the NCO nanomaterials. The diffraction patterns were recorded in the 2θ range 10 − 80° with a step size of 0.05°. The surface morphology and microstructure were examined by field emission scanning electron microscopy (FESEM; Nova SEM 200, FEI) and scanning transmission electron microscopy (STEM; TALOS F200X, FEI) equipped with energy-dispersive X-ray spectroscopy (EDS). The surface valence state was analyzed by X-ray photoelectron spectroscopy (XPS; PHI 5000 Versa Probe II, ULVAC PHI) using Al Kα radiation (hν = 1486.6 eV). The binding energies were calibrated using the C 1 s peak at 284.6 eV as the reference. Fourier Transform Infrared (FT-IR) spectra were recorded using a Jasco-FTIR-6300 spectrometer in the wavenumber range 1500–400 cm^−1^ after preparing the samples on KBr pellets. Raman spectra were also obtained using a Raman spectrometer (Horiba Co., Japan) with a He–Ne laser (632.8 nm) as the excitation source. Brunauer–Emmett–Teller (BET; BELSORP mini II, MicrotracBEL, Corp.) low-temperature N_2_ adsorption–desorption isotherms were measured using a BELSORP mini II analyzer (MicrotracBEL Corp.) to evaluate the specific surface area and pore size distribution.

### Electrochemical measurements

All the electrochemical measurements, such as cyclic voltammetry (CV) and chronoamperometry (CA) were performed on a PGSTAT302N potentiostat (Metrohm-Autolab) using a three-electrode system in 0.1 M aqueous NaOH solution at room temperature. A glassy carbon electrode (GC)-based working electrode, an Ag/AgCl electrode, and a platinum plate were used as the working, reference, and counter electrodes, respectively. The CVs were recorded between 0 and 0.6 V at different scanning rates of 5–100 mV s^−1^. To measure the ECSA, the CVs were conducted in the range of 0.1–0.2 V at different scan rates (5–100 mV s^−1^). The CA response of the samples to glucose was obtained at 0.5 V under stirring. For sensitivity and selectivity measurements, 0.01–6 mM glucose solutions, 0.1 mM LA, DA, AA, and UA were used in 0.1 M NaOH solution. The reproducibility of the UNCO was examined by three different electrodes with addition of 5 mM glucose at the optimal condition. The repeatability was also examined by three times of measurements by a single UNCO electrode in 6 h.

## Supplementary Information

Supplementary Information.

## Data Availability

All data generated or analyzed during this study are included in this published article (and its Supplementary Information files).
