# Supplementary material for: Synthetic control of the surface area in nickel cobalt oxide for glucose detection via additive-assisted wet chemical method
Source: Sci Rep. 2022 Nov 15;12:19546. doi: 10.1038/s41598-022-20859-4 (PMC9666531; doi:10.1038/s41598-022-20859-4)
Supplement: Supplementary file 1 — Supplementary Information. [file 41598_2022_20859_MOESM1_ESM.docx]

Supporting Information

**Synthetic control of the surface area in nickel cobalt oxide for glucose detection via additive-assisted wet chemical method**

*Kyu-bong Jang^a,†^, Kyoung Ryeol Park^b,†^, Chan Bin Mo^a^, Seongtak Kim^a^, Jaeeun Jeon^c^, Sung-chul Lim^c^, Chisung Ahn^c^, HyukSu Han^d^, Dongju Kim^e^, Seung Hwan Lee^f,*^, Kang min Kim^a,*^, and Sungwook Mhin^e,*^*

^a^ Korea Institute of Industrial Technology, 137-41 Gwahakdanji-ro, Gangneung, 25440, Republic of Korea

^b^ Korea Institute of Industrial Technology, 55, Jongga-ro, Jung-gu, Ulsan, 44413, Republic of Korea

^c^ Korea Institute of Industrial Technology, 156 Gaetbeol-ro, Incheon, 21999, Republic of Korea

^d^ Department of Energy Engineering, Konkuk University, 120 Neungdong-ro, Seoul, 05029, Republic of Korea

^e^ Department of Advanced Materials Engineering, Kyonggi University, 154-42 Gwanggyosan-ro, Suwon, 16227, Republic of Korea

^f^ School of Mechanical Engineering, Hanyang University, 04763, Republic of Korea

† These authors contributed equally to this work.

* Correspondence and request for materials should be addressed to K. M. Kim (kmkim@kitech.re.kr) or S. Lee ([seunghlee@hanyang.ac.kr](mailto:seunghlee@hanyang.ac.kr)) or S. Mhin ([swmhin@kgu.ac.kr](mailto:swmhin@kgu.ac.kr)).

**Figure S1.** FE-SEM image of the NCO nanomaterials. The different nanostructures are also shown.

**Figure S2.** EDS mapping image of Ni, Co, and O in the NCO nanomaterials.

**Figure S3.** The XPS spectra of Ni2p and Co2p; (a) PNCO, (b) TNCO, and (c) FNCO

**Figure S4.** The XPS spectra of O1s; (a) UNCO, (b) PNCO, (c) TNCO, and (d) FNCO

**Figure S5.** FE-SEM image of the as-prepared NCO nanomaterials.

**Figure S6.** XRD patterns of the as-prepared NCO nanomaterials; (a) as-prepared UNCO, (b) as-prepared PNCO, (c) as-prepared TNCO, and (d) as-prepared FNCO.

**Figure S7.** FE-SEM image of the as-prepared NCO nanomaterials.

**Figure S8.** The XPS spectra of Ni2p and Co2p of the as-prepared NCO nanomaterials.

**Figure S9.** The XPS spectra of C1s, N1s, and O1s of the as-prepared NCO nanomaterials.

**Figure S10.** Cyclic Voltammetry curves of (a) UNCO, (b) PNCO, (c) TNCO, and (d) FNCO electrodes at different scan rates in 0.1M NaOH solution.

**Figure S11.** Cyclic voltammetry curve in 0.1 M NaOH for (a) UNCO, (b) PNCO, (c) TNCO, and (d) FNCO, in the non-faradic region of 0.10-0.20 V vs. Ag/AgCl at various scan rates of 5-100 mV s^-1^. The measured capacitive currents are plotted as a function of scan rate.

**Figure S12.** The calibration curve between glucose concentration (C) and current response of the NCO nanomaterials. The sensitivity can be calculated as the ratio of the slope to electrode area. (a) UNCO; I(μA) = (32.88 ± 0.63)C + (4.87 ± 1.66) (R^2^=0.993), (b) PNCO; I(μA) = (29.24 ± 0.99)C + (9.26 ± 2.63) (R^2^=0.99), (c) TNCO, I(μA) = (27.05 ± 0.53)C + (4.97 ± 1.41) (R^2^=0.99), and (d) FNCO, I(μA) = (27.21 ± 0.82)C + (9.20 ± 2.17) (R^2^=0.992).

**Figure S13.** Chronoamperometry response of (a) UNCO, (b) PNCO, (c) TNCO, and (d) FNCO electrodes for stability in 0.1 M NaOH. The stability of the nanomaterials was examined by a chronoamperometry response under alkaline conditions (0.1 M NaOH) containing 1 mM for an extended period of time (80,000 s).

**Figure S14.** Morphological and structural characterizations of UNCO after 24 h of chronoamperometry at 0.5 V in an electrolyte containing 0.1 M NaOH and 1 mM glucose. (a) TEM images along with the SAED patterns, lattice-resolved HRTEM image, and corresponding HADDF images of Ni, Co, and O. (b) The XPS spectra of Ni2p, Co2p, and O1s.

**Figure S15.** (a) Long-term stability, (b) reproducibility, and (c) repeatability of UNCO electrode.

**Figure S16.** Schematic illustration of electro-paths on the morphology-controlled NiCo_2_O_4_ nanostructure

**Table S1.** Electrochemical performance of NCO in our work to that of non-enzymatic glucose sensors in other works.

**Table S2.** Synthetic details of NiCo2O4 (NCO) with controlled morphology via additive-assisted hydrothermal synthesis. For controlling the morphology of NCO, additives such as urea, hexamethylene-tetramine (HMT), and ammonium fluoride (NH4F) were selectively added in the above solution.

# Figure S1


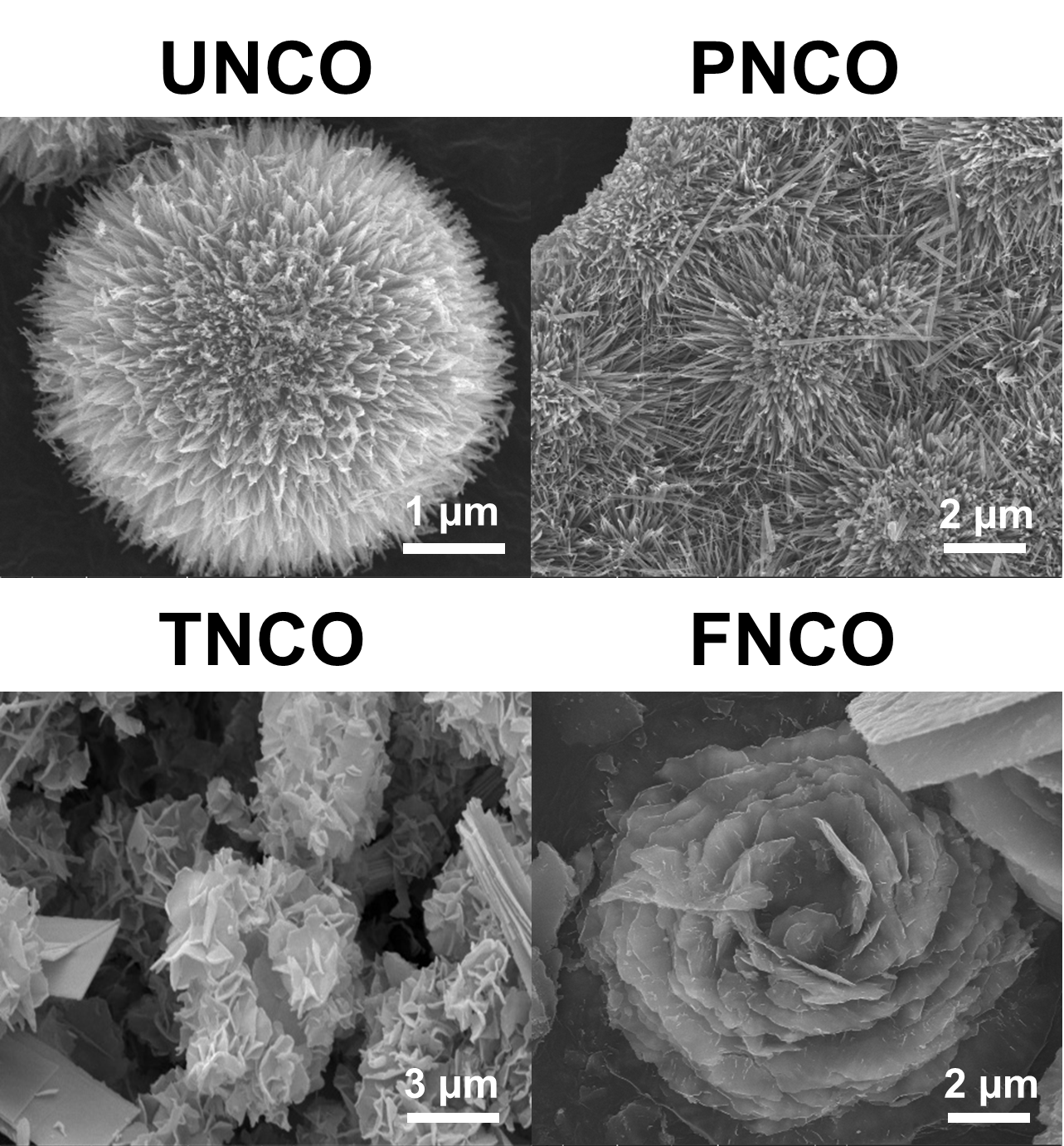


**Figure S1.** FE-SEM image of the NCO nanomaterials. The different nanostructures are also shown.

# Figure S2

**
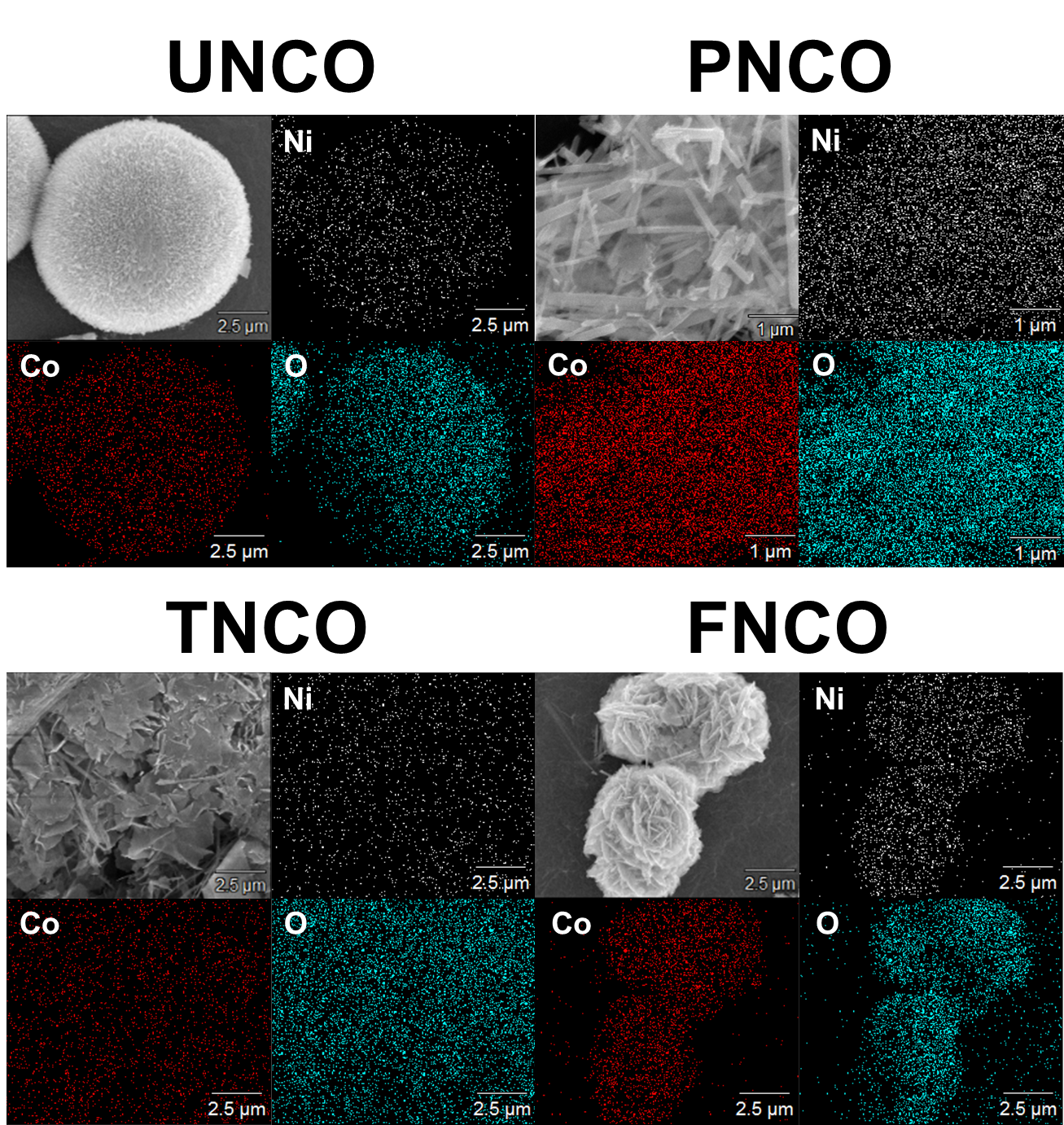
**

**Figure S2.** EDS mapping image of Ni, Co, and O in the NCO nanomaterials.

# Figure S3


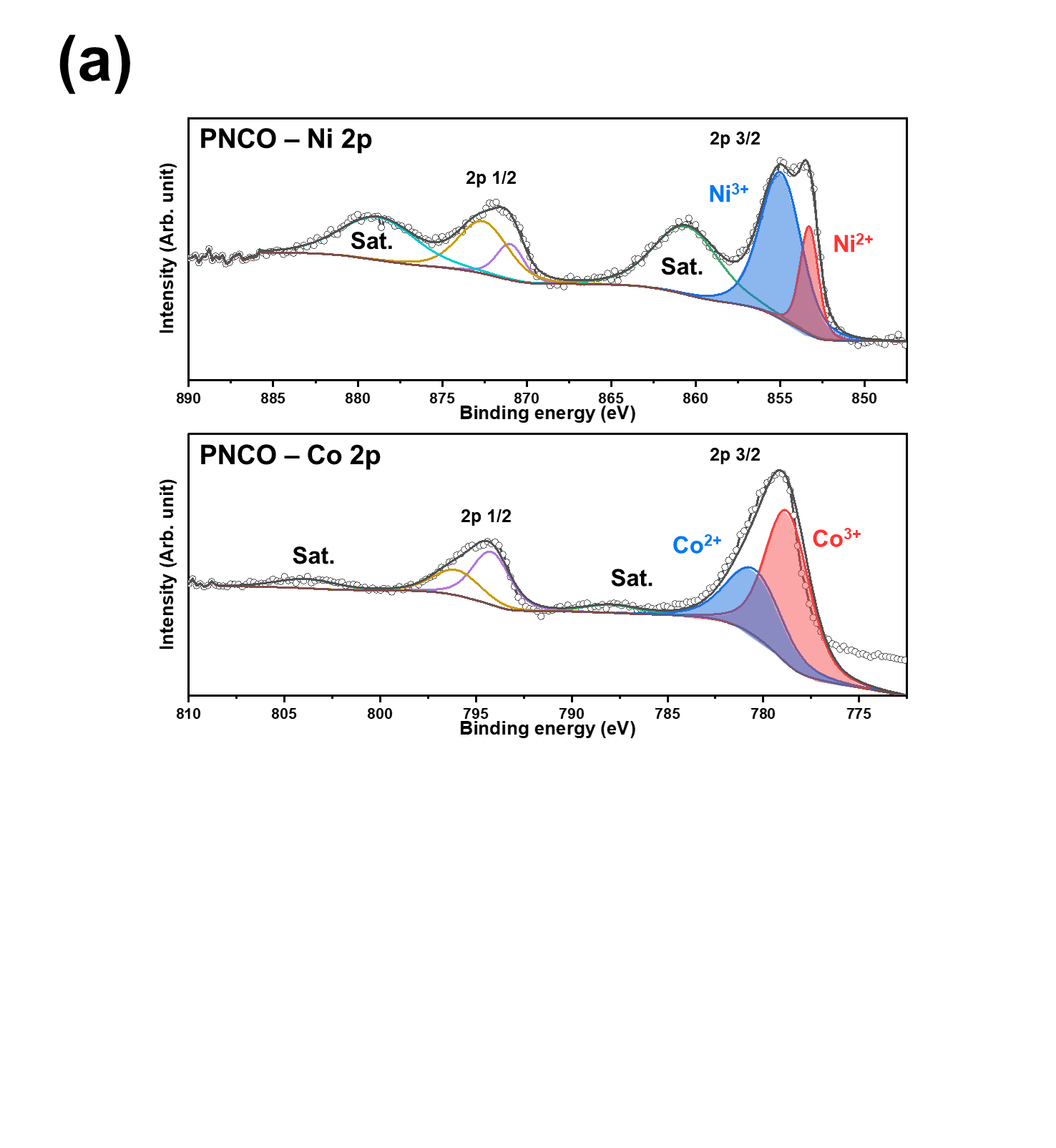


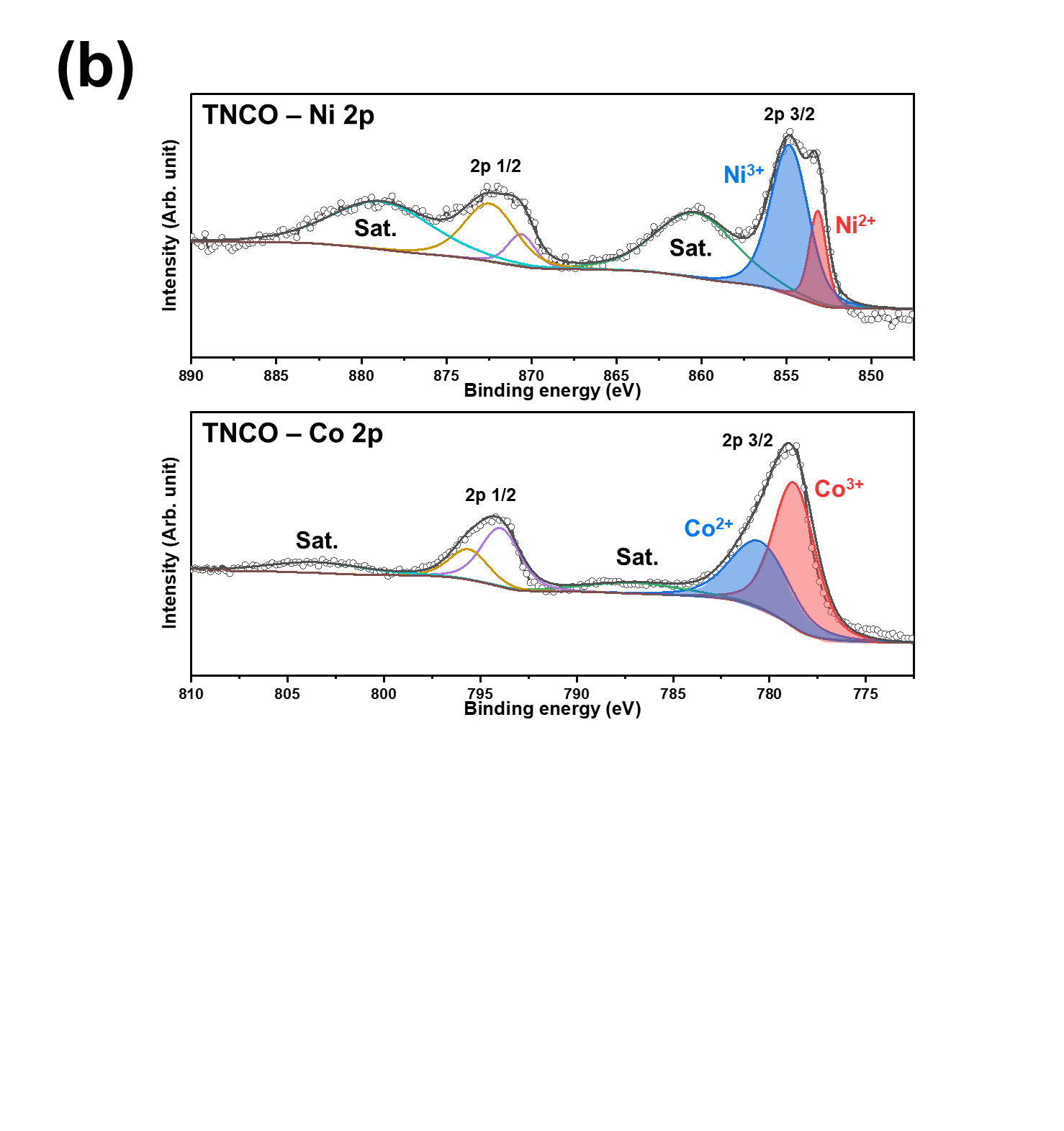


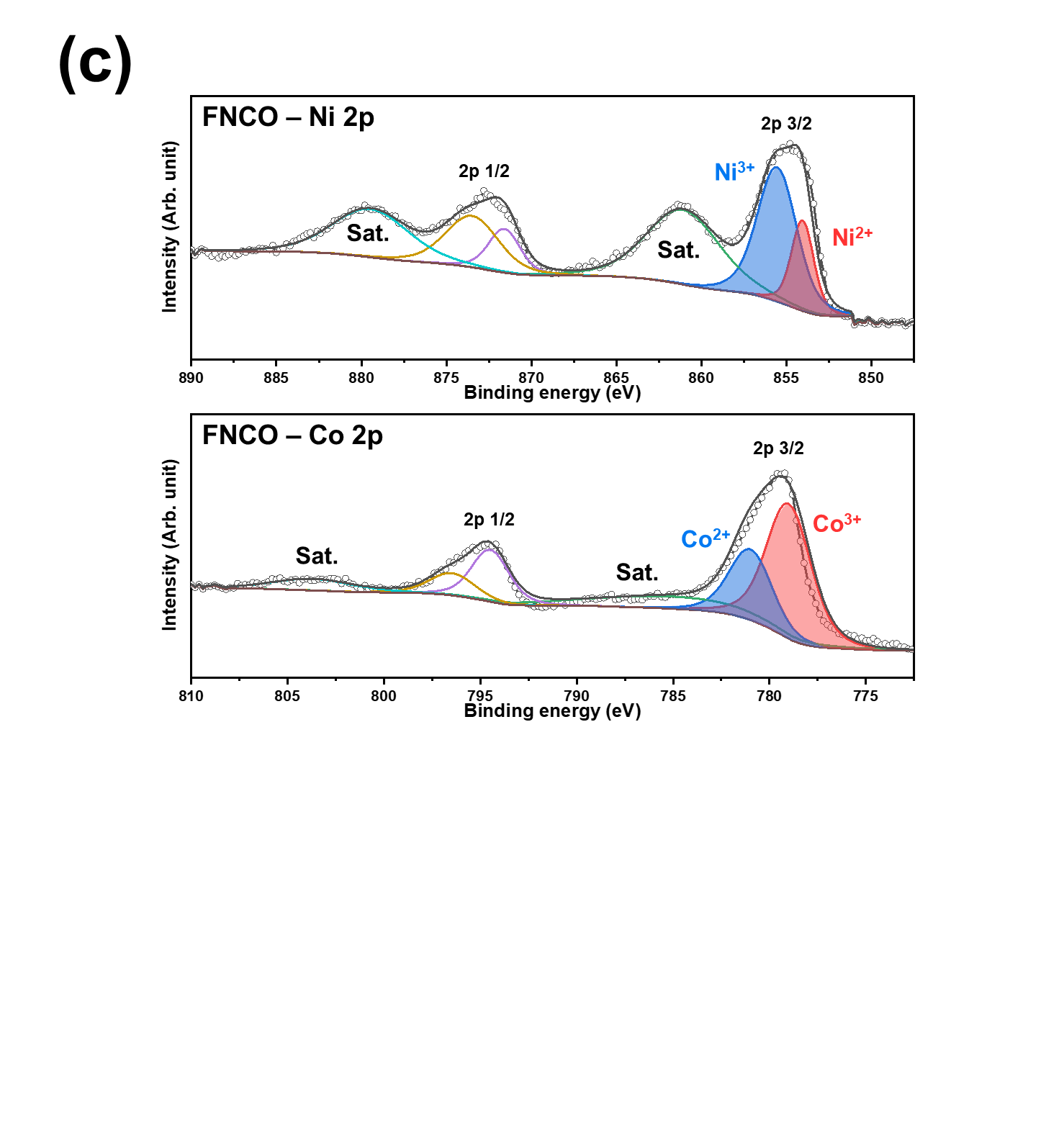


**Figure S3.** The XPS spectra of Ni2p and Co2p; (a) PNCO, (b) TNCO, and (c) FNCO

# Figure S4


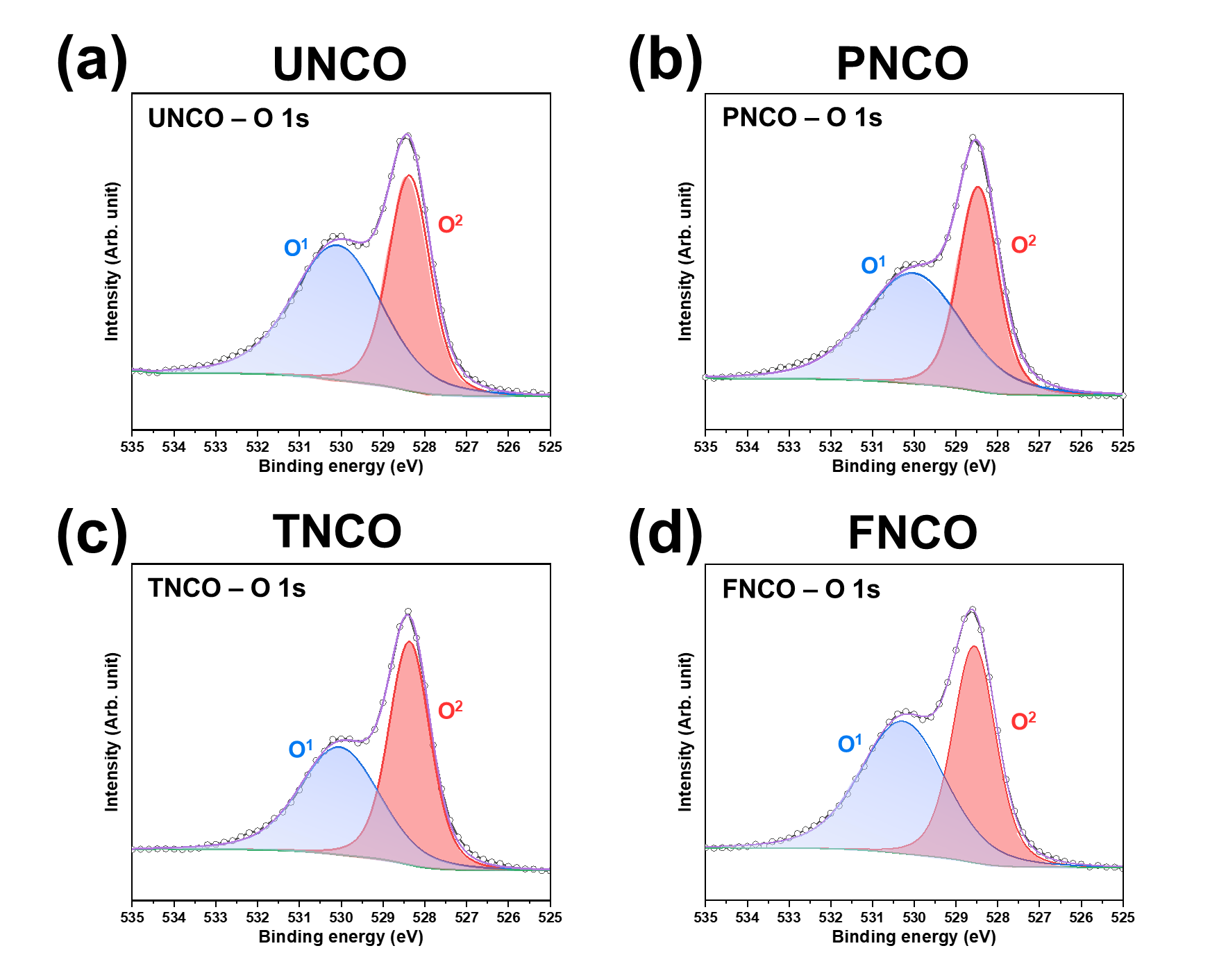


**Figure S4.** The XPS spectra of O1s; (a) UNCO, (b) PNCO, (c) TNCO, and (d) FNCO

# Figure S5


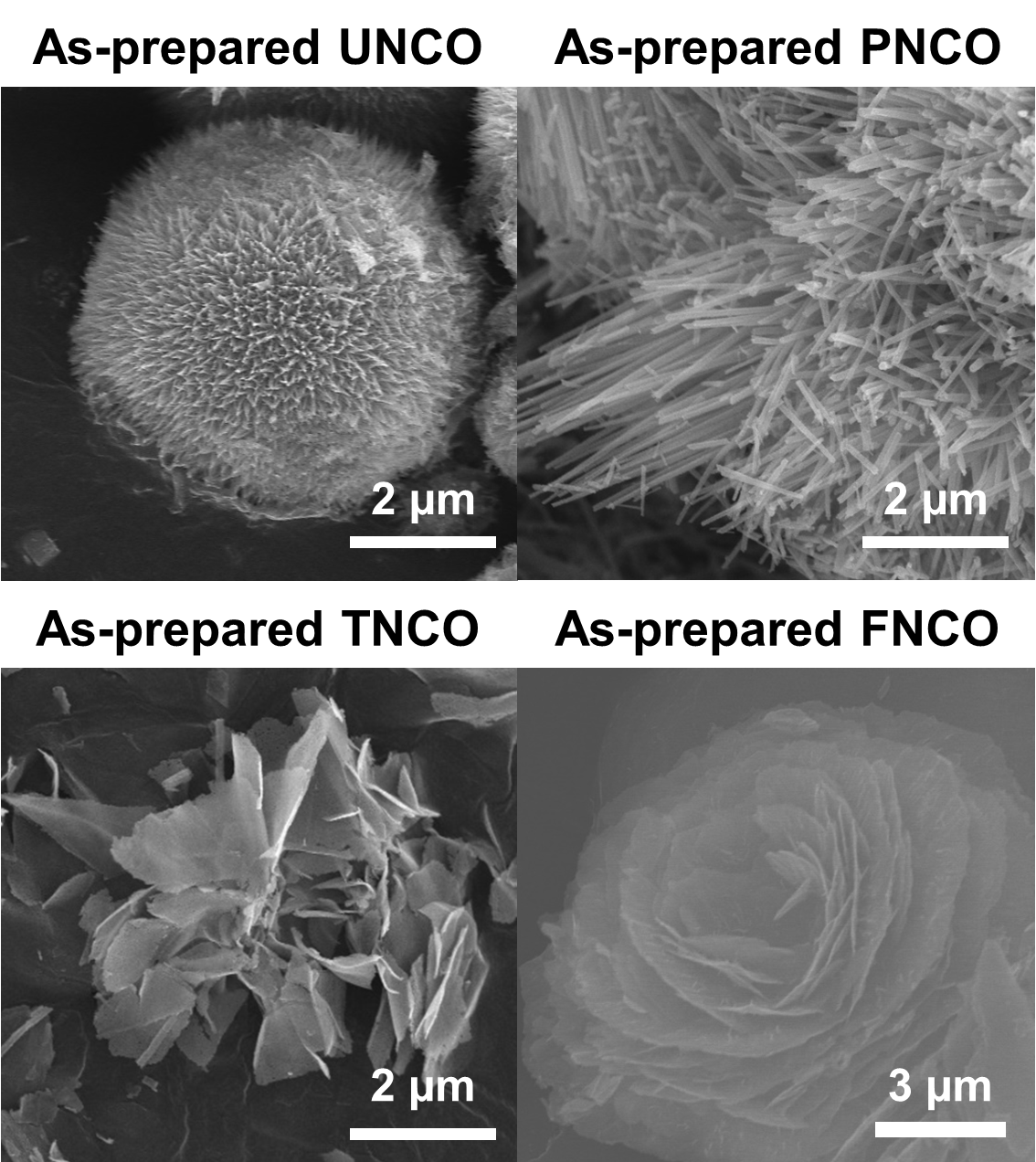


**Figure S5.** FE-SEM image of the as-prepared NCO nanomaterials.

The chemical reactions involved in the formation of as-prepared UNCO and PNCO are given below :

CO(NH_2_)_2_ + H_2_O → 2 NH_3_ + CO_2_

CO_2_ + H_2_O → CO_3_^2-^ + 2 H^+^

NH_3_ + H_2_O → NH_4_^+^ + OH^-^

3 Ni^2+^ + CO_3_^2-^ + 2 OH^-^ + 4 H_2_O → Ni_2_(CO_3_)(OH)_2_ ∙ 4 H_2_O

Co^2+^ + 0.11 H_2_O + OH^-^ + 0.5 CO_3_^2-^ → Co(CO_3_)_0.5_(OH)_0.11_ ∙ H_2_O

The chemical reactions involved in the formation of as-prepared TNCO and FNCO are given below :

(CH_2_)_6_N_4_ + 6 H_2_O → 4 NH_3_ + 6 HCHO

NH_3_ + H_2_O → NH_4_^+^ + OH^-^

Ni^+^ + 2 OH^-^ → Ni(OH)_2_

Co^2+^ + 2 OH^-^ → Co(OH)_2_

# Figure S6


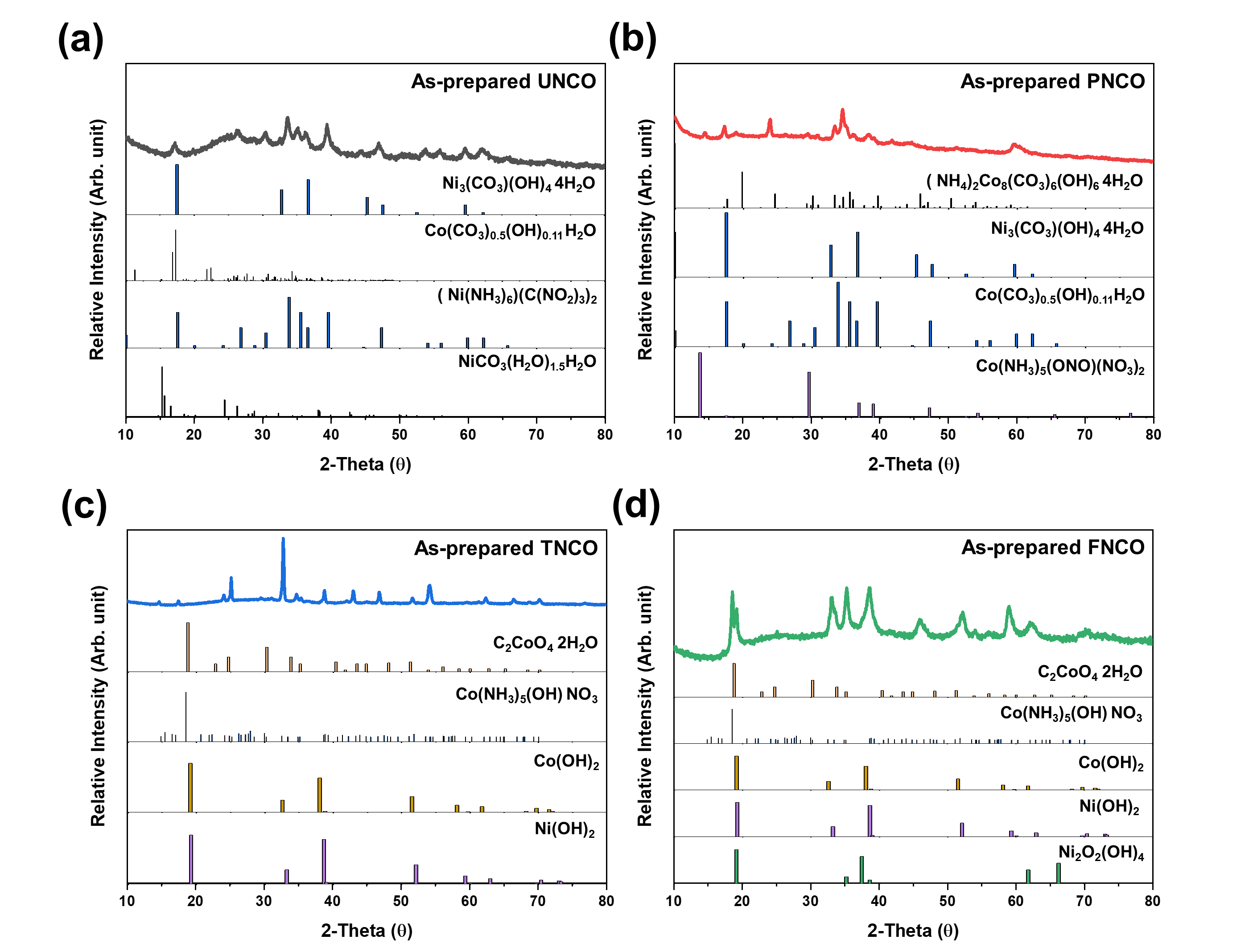


**Figure S6.** XRD patterns of the as-prepared NCO nanomaterials; (a) as-prepared UNCO, (b) as-prepared PNCO, (c) as-prepared TNCO, and (d) as-prepared FNCO.

# Figure S7


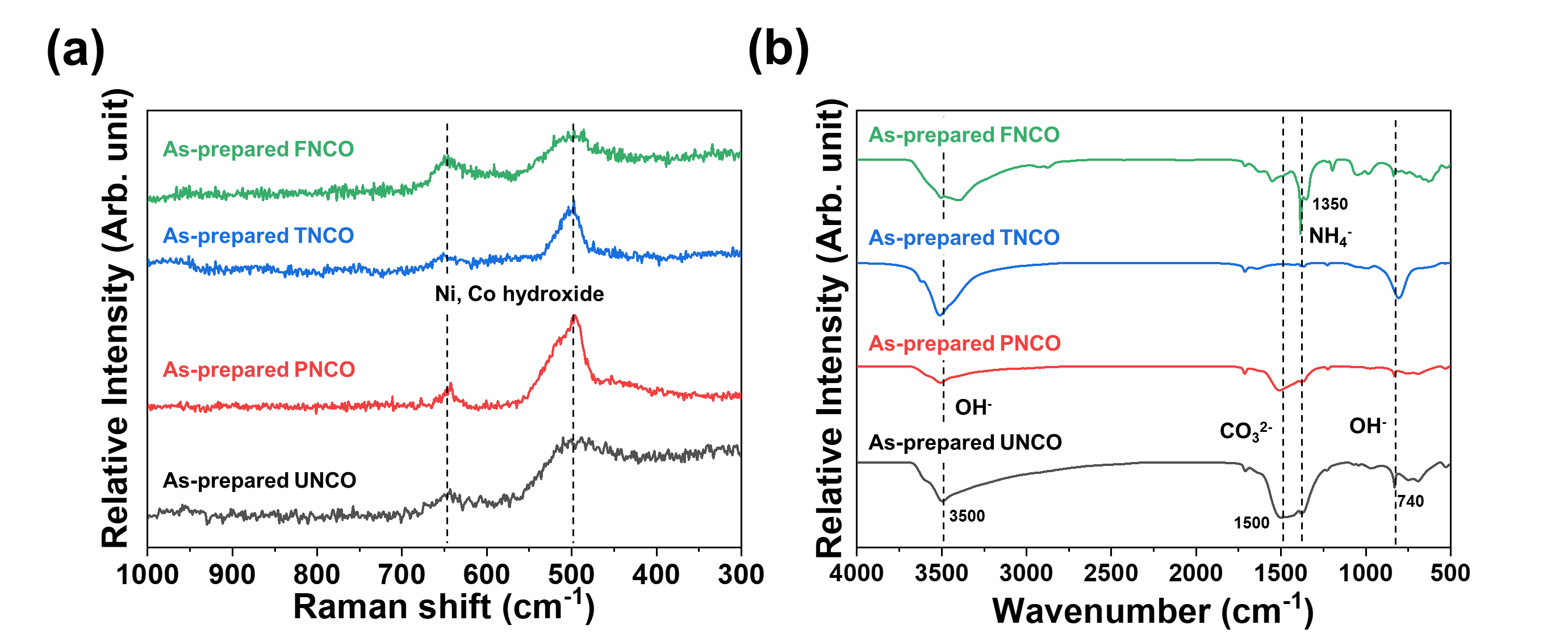


**Figure S7.** (a) Raman spectra, (b) FT-IR spectra of the as-prepared NCO nanomaterials.

# Figure S8


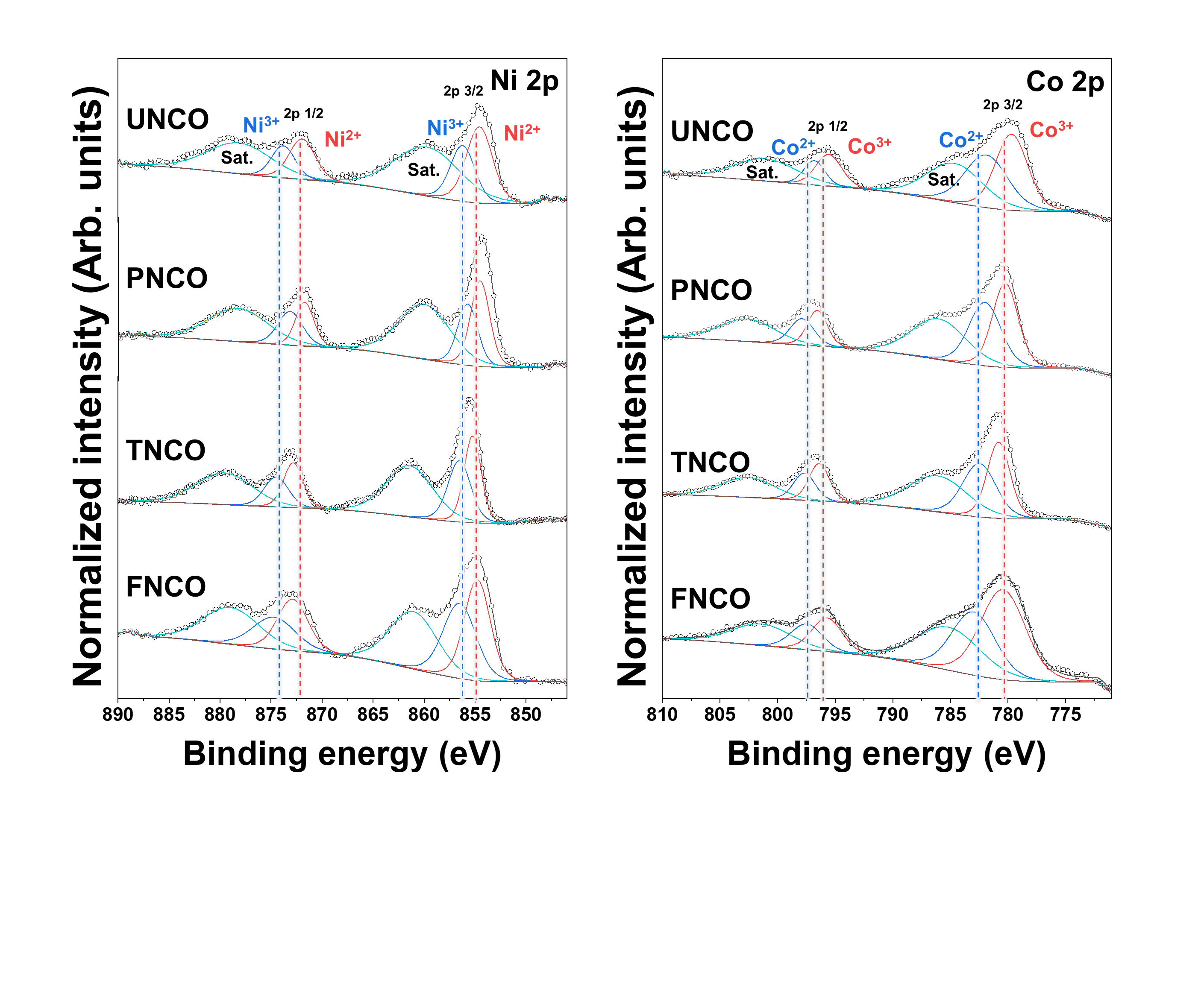


**Figure S8**. The XPS spectra of Ni2p and Co2p of the as-prepared NCO nanomaterials.

# Figure S9


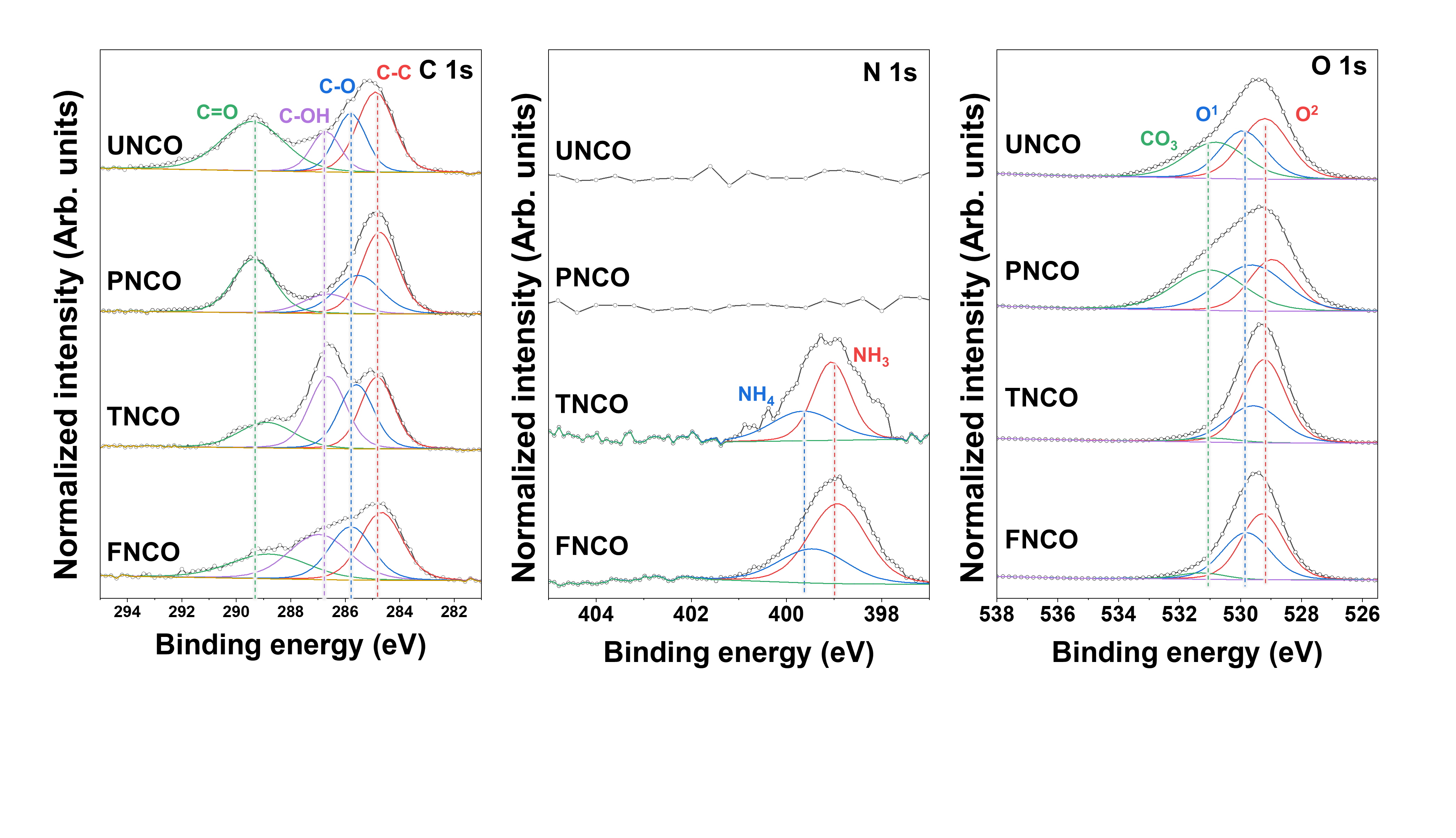


**Figure S9.** The XPS spectra of C1s, N1s, and O1s of the as-prepared NCO nanomaterials.

# Figure S10


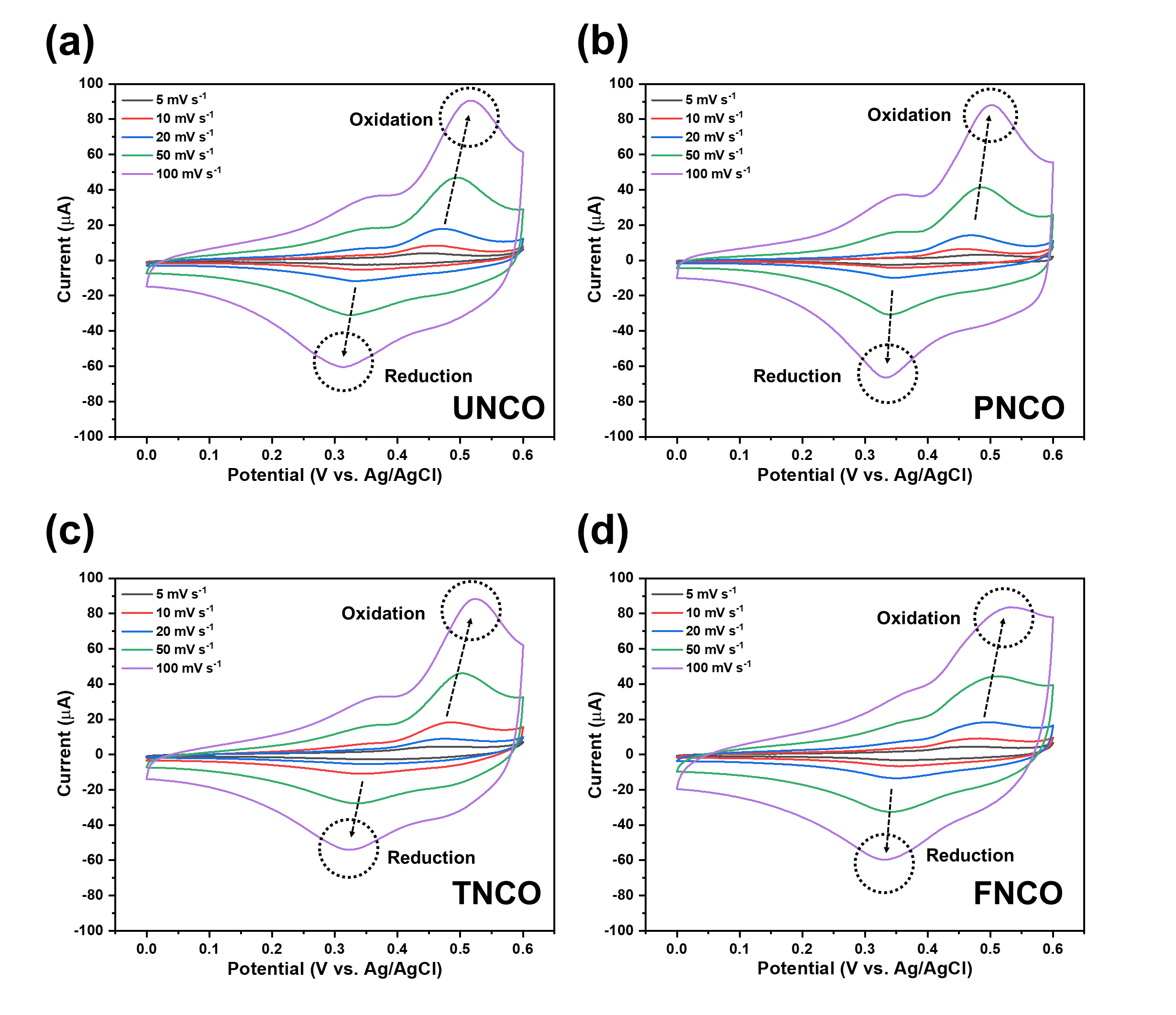


**Figure S10.** Cyclic Voltammetry curves of (a) UNCO, (b) PNCO, (c) TNCO, and (d) FNCO electrodes at different scan rates in 0.1M NaOH solution.

# Figure S11


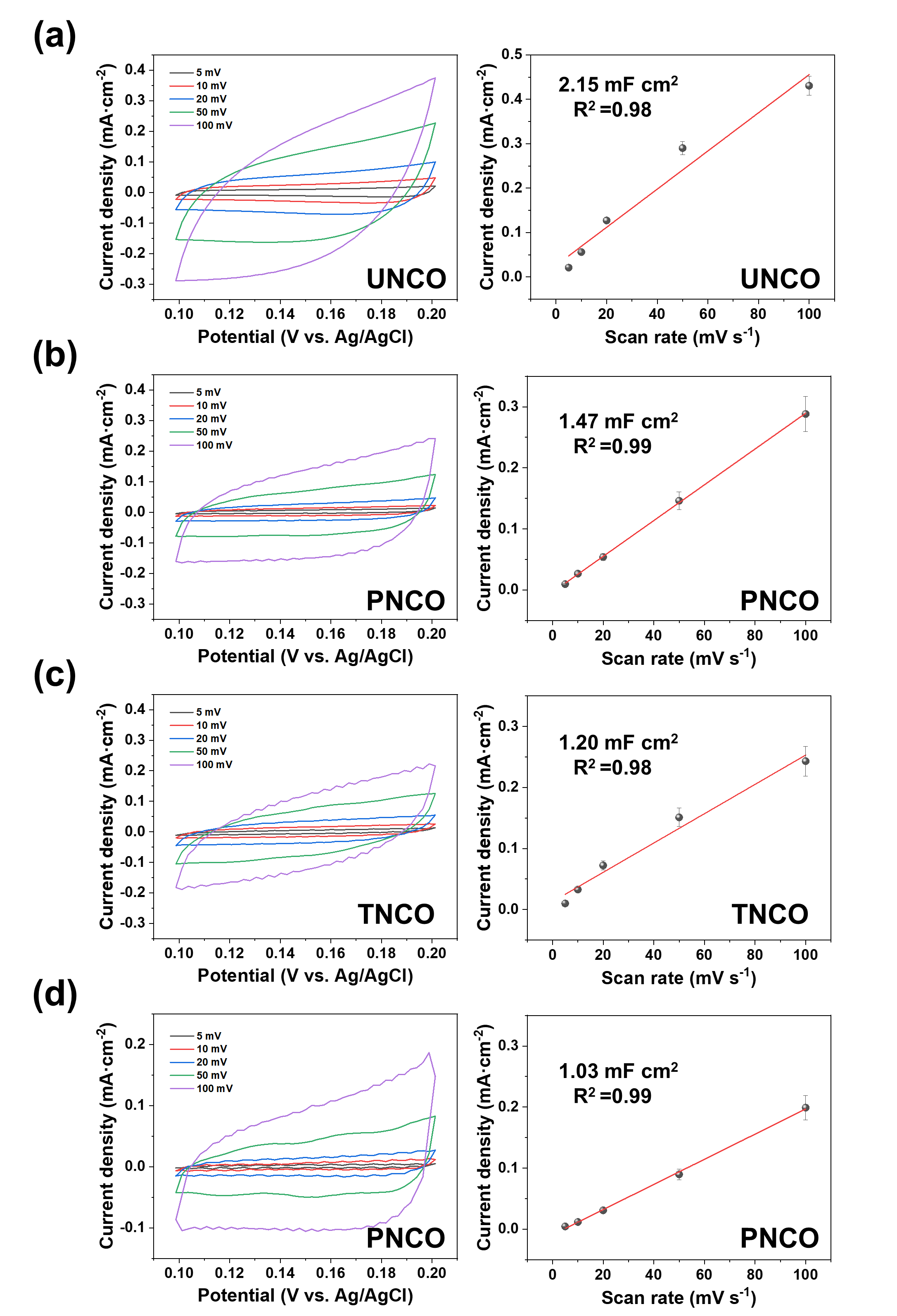


**Figure S11.** Cyclic voltammetry curve in 0.1 M NaOH for (a) UNCO, (b) PNCO, (c) TNCO, and (d) FNCO, in the non-faradic region of 0.10-0.20 V vs. Ag/AgCl at various scan rates of 5-100 mV s^-1^. The measured capacitive currents are plotted as a function of scan rate. The ECSA was measured using the formula, ECSA = Cdl /Cs, where Cs is the specific capacitance of the sample or the capacitance of an atomically smooth planar surface of the material per unit area under identical electrolyte conditions (Cs = 0.04 mF cm^−2^ in 0.1 M NaOH).

# Figure S12


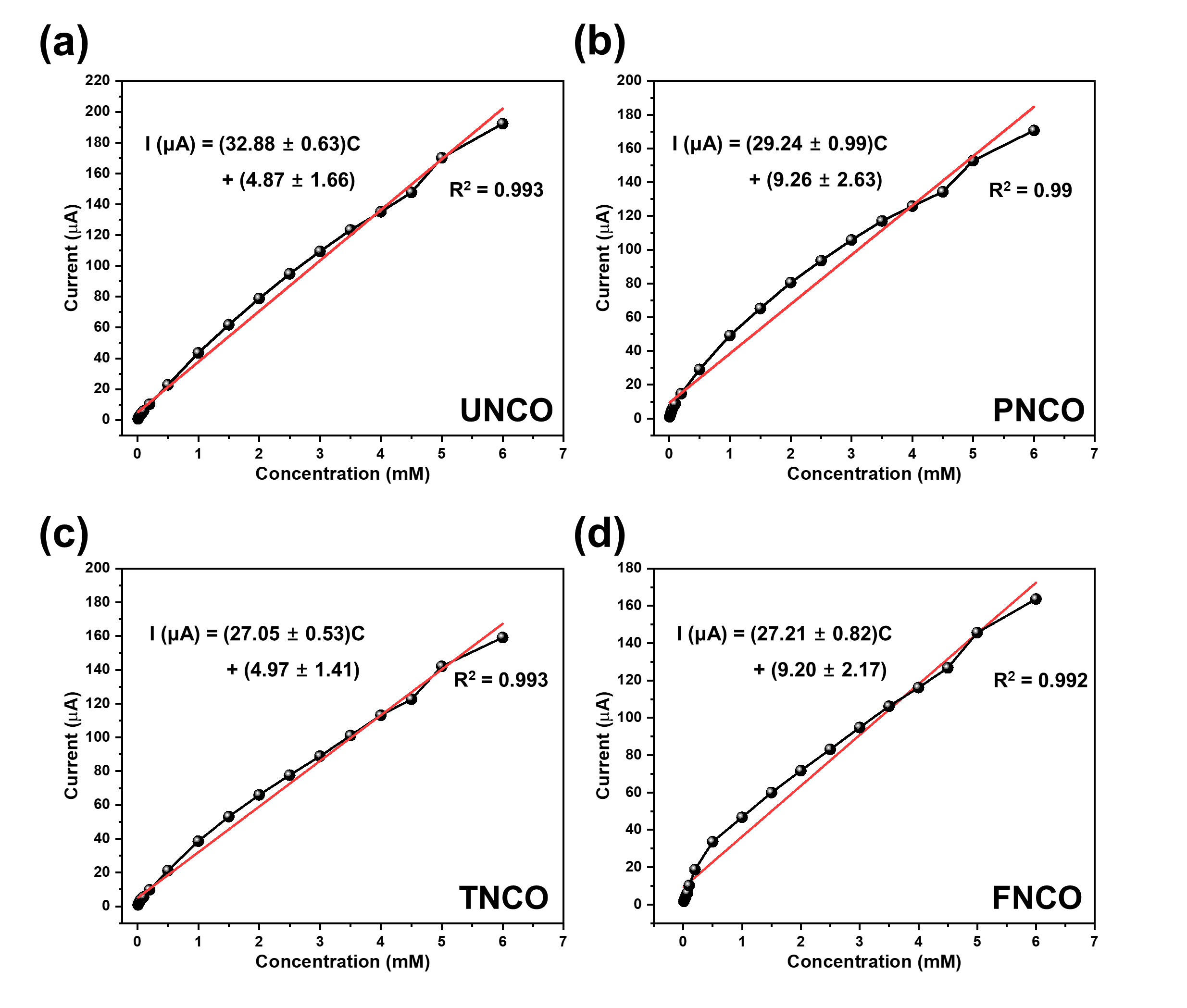


**Figure S12.** The calibration curve between glucose concentration (C) and current response of the NCO nanomaterials. The sensitivity can be calculated as the ratio of the slope to electrode area. (a) UNCO; I(μA) = (32.88 ± 0.63)C + (4.87 ± 1.66) (R^2^=0.993), (b) PNCO; I(μA) = (29.24 ± 0.99)C + (9.26 ± 2.63) (R^2^=0.99), (c) TNCO, I(μA) = (27.05 ± 0.53)C + (4.97 ± 1.41) (R^2^=0.99), and (d) FNCO, I(μA) = (27.21 ± 0.82)C + (9.20 ± 2.17) (R^2^=0.992).

# Figure S13


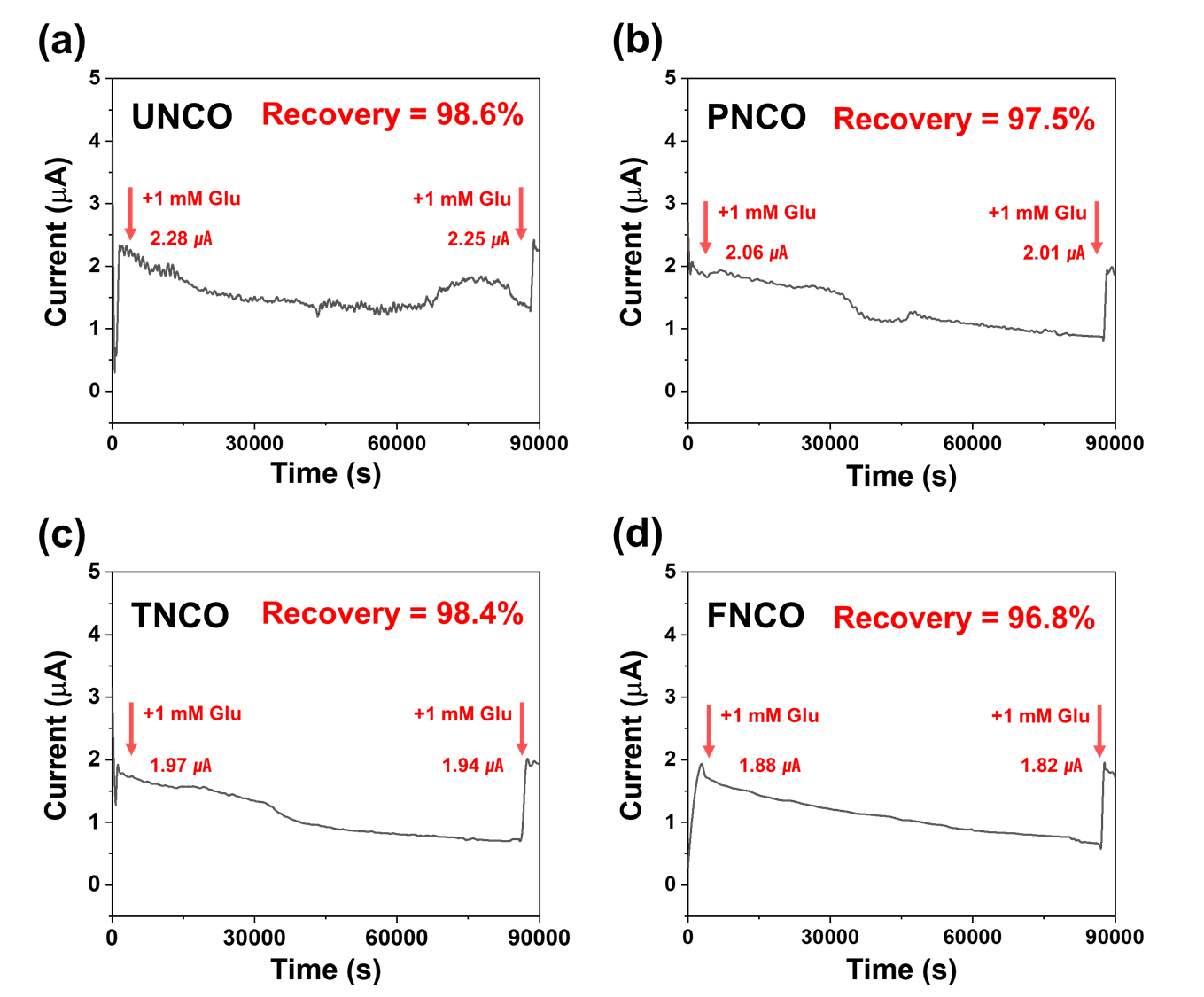


**Figure S13.** Chronoamperometry response of (a) UNCO, (b) PNCO, (c) TNCO, and (d) FNCO electrodes for stability in 0.1 M NaOH. The stability of the nanomaterials was examined by a chronoamperometry response under alkaline conditions (0.1 M NaOH) containing 1 mM for an extended period of time (80,000 s).

# Figure S14


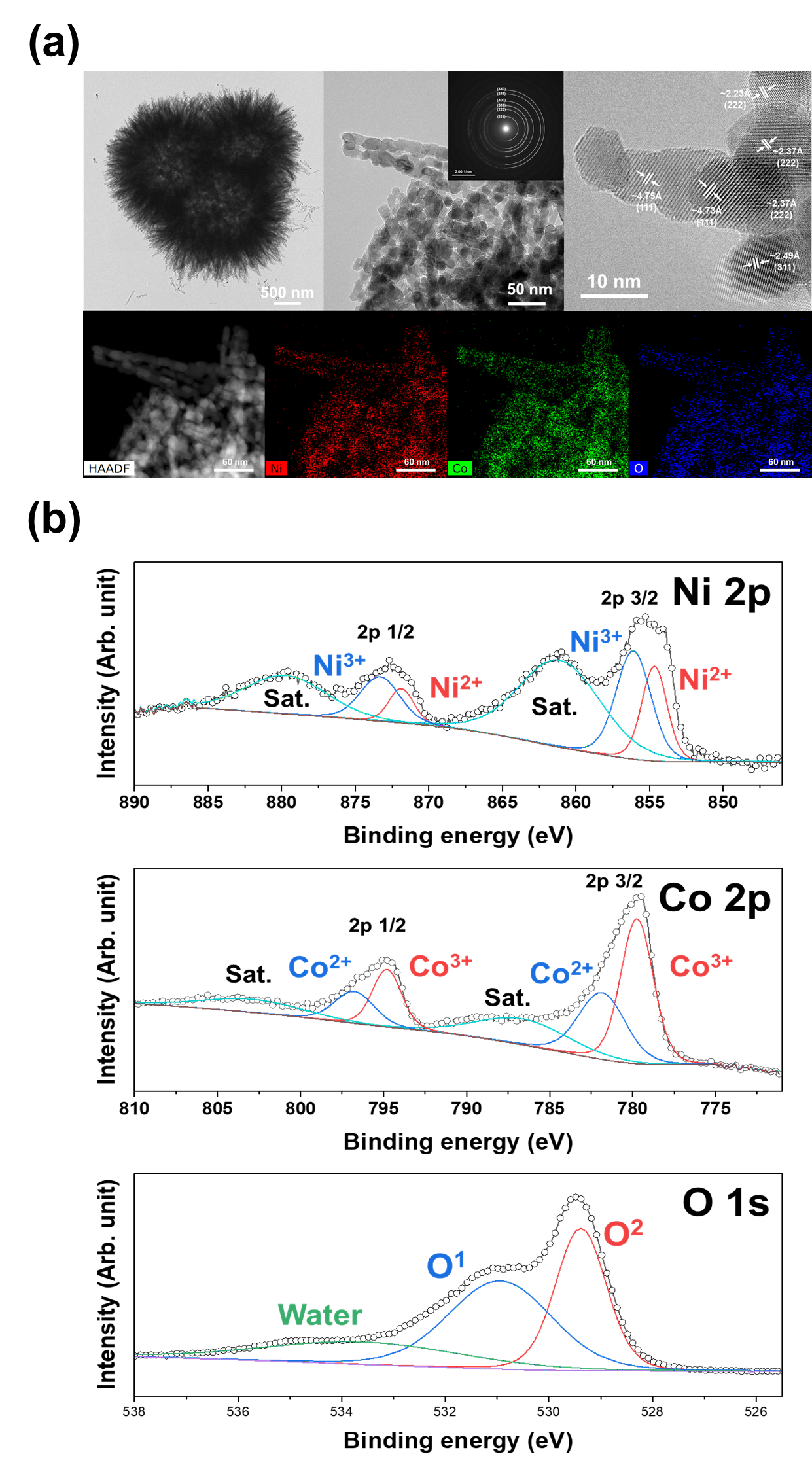


**Figure S14.** Morphological and structural characterizations of UNCO after 24 h of chronoamperometry at 0.5 V in an electrolyte containing 0.1 M NaOH and 1 mM glucose. (a) TEM images along with the SAED patterns, lattice-resolved HRTEM image, and corresponding HADDF images of Ni, Co, and O. (b) The XPS spectra of Ni2p, Co2p, and O1s.

# Figure S15


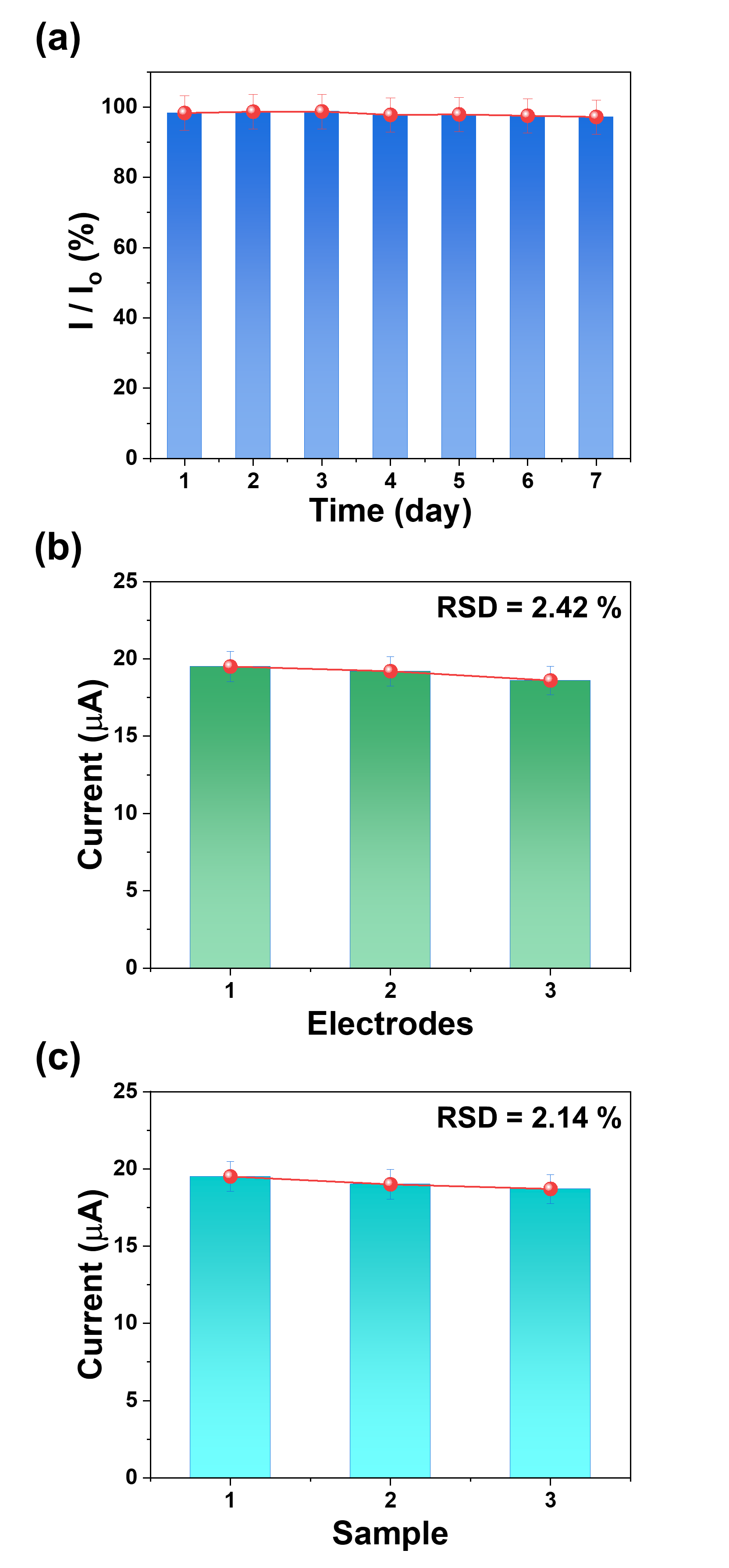


**Figure S15.** (a) Long-term stability, (b) reproducibility, and (c) repeatability of UNCO electrode. The long-term stability of the UNCO was examined by a chronoamperometry response under alkaline conditions (0.1 M NaOH) containing 5 mM for 7 days. The reproducibility of the UNCO was examined by three different electrodes with addition of 5 mM glucose at the optimal condition. The repeatability was also examined by three times of measurements by a single UNCO electrode in 6 h.

# Figure S16


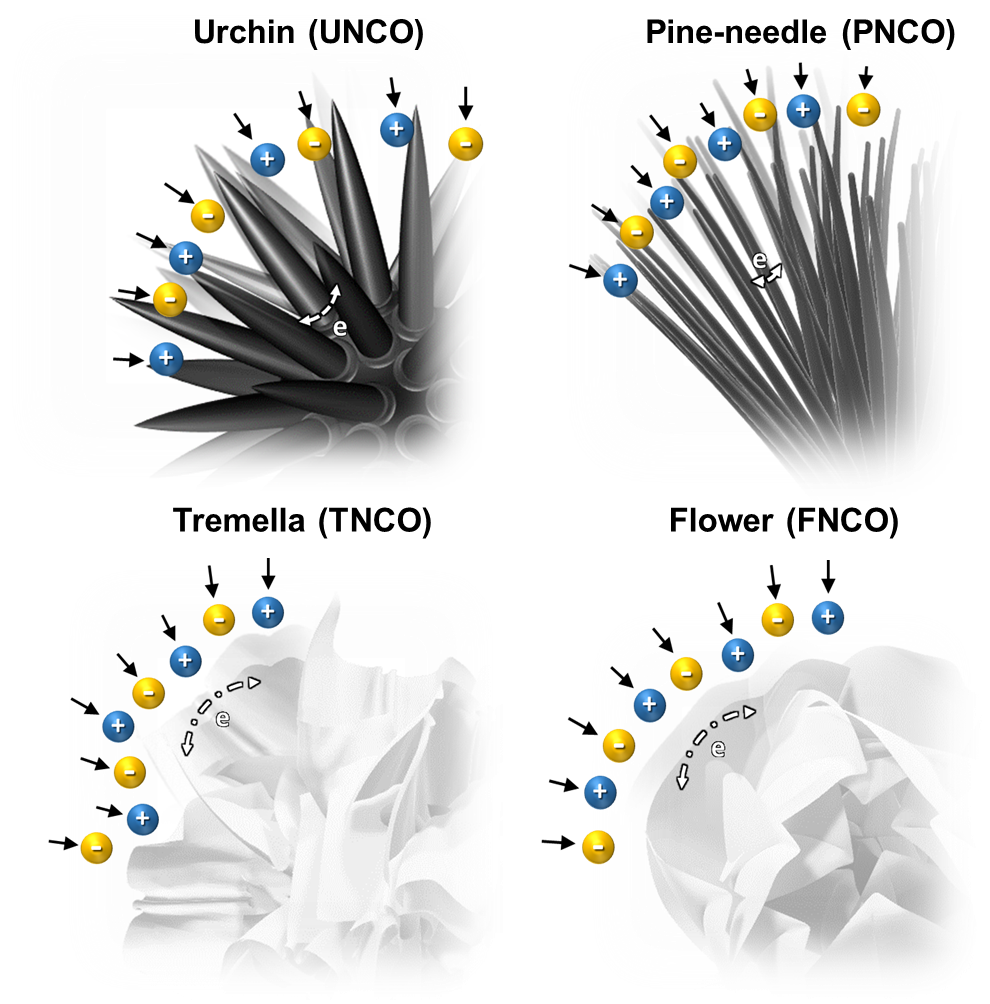


**Figure S16.** Schematic illustration of electropaths on the morphology-controlled NiCo_2_O_4_ nanostructure.

# Table S1

**Table S1.** Electrochemical performance of NCO in our work to that of non-enzymatic glucose sensors in other works.

| **Electrode** | **Method** | **Sensitivity** | **Linear range** | **Correlation**  **Coefficient** | **Detection**  **limit** | **Refs.** |
| --- | --- | --- | --- | --- | --- | --- |
|  |  | **(μAmM^-1^ cm^-2^)** | **(mM)** | **(R^2^)** | **(μM)** |  |
| **UNCO** | CA | 116.33 | 0.01 – 6 | 0.99 | 0.0783 | This work |
| **PNCO** | CA | 103.48 | 0.01 – 6 | 0.99 | 0.0623 | This work |
| **TNCO** | CA | 95.72 | 0.01 – 6 | 0.992 | 0.0673 | This work |
| **FNCO** | CA | 84.72 | 0.01 – 6 | 0.993 | 0.0723 | This work |
| **NCO/CNT** | CA | 66.15 | 0.02 – 12.12 | 0.99 | 5 | [S1] |
| **CuCo_2_O_4_** | CA | 3.625 | Up to 0.32 | - | 5 | [S2] |
| **NiO** | CA | 32.91 | Up to 1.94 | - | 1.28 | [S3] |
| **Co_3_O_4_** | CA | 36.25 | Up to 2.04 | - | 0.97 | [S4] |

The LOD (Limit of detection) calculated by the formula in term of LOD = 3𝜎/𝑏, where 𝜎 is the standard deviation of background which is obtained by measuring the current response of NCO electrode in the 0.1M NaOH solution without glucose, and 𝑏 is the sensitivity of the NCO.

[S1] L. Wang, X. Lu, Y. Ye, L. Sun, Y. Song, *Electrochim. Acta*. **114,** (2016) 484.

[S2] P. K. Kannan, C. Hu, H. Morgan, C. S. Rout, *Chem. Asian. J*. **11,** (2016) 1837.

[S3] S. Liu, K. S. Hui, K. N. Hui, *ACS Appl. Mater. Interfaces.* **8,** (2016) 3258.

[S4] Y. Ding, Y. Wang, L. Zhang, H. Zhang, Y. Lei, *J. Mater. Chem*. **22,** (2012) 980.

# Table S2

**Table S2.** Synthetic details of NiCo_2_O_4_ (NCO) with controlled morphology via additive-assisted hydrothermal synthesis. For controlling the morphology of NCO, additives such as urea, hexamethylene-tetramine (HMT), and ammonium fluoride (NH_4_F) were selectively added in the above solution.

| **Material** | **Sample** | **Morphology** | **Urea** | **HMT** | **NH_4_F** | **Hydrothermal** | **Heat**  **treatment** | |
| --- | --- | --- | --- | --- | --- | --- | --- | --- |
|  |  |  | **(mmol)** | **(mmol)** | **(mmol)** |  |  |  |
| NiCo_2_O_4_ | UNCO | Urchin | 12 | - | - | 120 ℃  6 h | 400 ℃  4 h |  |
|  | PNCO | Pine-needle | 12 | - | 6 |  |  |  |
|  | TNCO | Tremella | - | 12 | - |  |  |  |
|  | FNCO | Flower | - | 12 | 6 |  |  |  |
